# Training and provision of mobility aids to promote autonomy and mobility of older patients in a geriatric emergency department: A protocol for a randomized controlled trial

**DOI:** 10.1371/journal.pone.0304397

**Published:** 2024-07-31

**Authors:** Fernanda Sato Polesel, Sâmia Denadai, Márlon Juliano Romero Aliberti, Christian Valle Morinaga, Mario Chueire de Andrade-Junior, Itiana Cardoso Madalena, Wellington Pereira Yamaguti, Pedro Kallas Curiati, Renato Fraga Righetti

**Affiliations:** 1 Hospital Sírio-Libanês, São Paulo, Brazil; 2 Research Institute, Hospital Sírio-Libanês, São Paulo, Brazil; 3 Laboratorio de Investigaçao Medica em Envelhecimento (LIM-66), Serviço de Geriatria, Hospital das Clinicas, Disciplina de Geriatria, Faculdade de Medicina, Universidade de São Paulo, Sao Paulo, Brazil; 4 Geriatric Emergency Department (ProAGE) Research Group, Hospital Sírio-Libanês, São Paulo, Brazil; UFPE: Universidade Federal de Pernambuco, BRAZIL

## Abstract

Older adults have higher rates of emergency department (ED) admissions when compared to their younger counterparts. Mobility is the ability to move around, but also encompasses the environment and the ability to adapt to it. Walking aids can be used to improve mobility and prevent falls. According to international guidelines, they must be available in Geriatric EDs. This study aims to evaluate the efficacy of a program of training and provision of walking aids (WA), associated or not with telemonitoring, on fear of falling, mobility, quality of life and risk of falls up to 3 and 6 months in older adults cared for in an ED. A randomized controlled trial will be carried out in the ED. Participants will be randomized and allocated into three groups, as follows: A) walking aid group will be trained for the use of a walking aid and receive guidance on safe gait; B) walking aid and telemonitoring group will receive training for the use of a walking aid, guidance on safe gait, and telemonitoring (every two weeks for first three months); C) Control group will receive only guidance on safe gait. Patients will undergo a baseline evaluation encompassing sociodemographic and clinical data, mobility in life spaces, gait speed, muscle strength, functionality, quality of life, fear of falling, history of falls, cognition and mood before the intervention. Gait time and fear of falling will be assessed again after the intervention in ED. Finally, mobility in life spaces, functionality, quality of life, fear of falling, history of falls, cognition, and mood will be assessed 3 and 6 months after discharge from the geriatric ED through a telephone interview. Provision of walking aids in the geriatric ED is currently recommended. This study will be the first randomized controlled trial that will evaluate the impact of training and provision of these devices in the ED.

**Trial registration number**: NCT05950269.

## Introduction

According to the National Center for Health Statistics, a department of the Centers for Disease Control and Prevention (CDC) in the United States, people aged 74 and over represented 56.7% of all emergency department (ED) visits in 2018 [1]. Indeed, the ED plays an important role for the older patient, constituting not only an emergency treatment center, but also a point of entry for highly complex acute care, continued health care services, and 24-hour care [2, 3]. According to American Geriatric ED Guidelines, proper care for older patients require policies, protocols, and flows designed for the specific needs of this population [3]. More recently, European geriatric ED guidelines have promoted the “5 Ms of Geriatrics”: mind, medication, multi-complexity, “most important” and mobility [4].

Older adult’s health is associated with psychological, social, and environmental factors and chronic diseases, which impact mobility, functionality, autonomy, quality of life, and activities of daily living (ADL) [5]. Mobility is a term used not only to describe a person’s physical ability to move, but also encompasses the person’s environment and ability to adapt to it [6]. It can be assessed by the individual’s ability to move from furniture (bed or chair), walk and climb stairs. Correlated with mobility, the life-space is an emerging concept related to functional, environmental, and social factors that define how people experience their daily lives [7]. Acutely ill older people who are admitted through the ED for complaints associated or not with falls have a higher risk of falls, with an impact on functionality, quality of life, cognition, return visits, and mortality [8]. Because of this, it is essential that screening and assessments of mobility, gait quality, and risk of falls for this older population be incorporated into ERs and to implement safety, mobility, and quality of life measures for this older population, linked to adequate and safe use of auxiliary devices for the gait.

Finally, new technologies have been developed to improve health care, including telemonitoring. Telemonitoring is defined as the use of information technology and telecommunications for remote health care [9] and its main advantages are reducing unnecessary visits and home visits, reducing the risk of infections, monitoring the rehabilitation process, and improving patient compliance [10]. However, most studies related to telemonitoring are carried out with patients who have chronic diseases. Also, there are no data on the effects of an intervention encompassing gait devices, associated or not with telemonitoring, to improve the mobility of older patients admitted to the ED despite recommendations from international societies and the Geriatric Emergency Department Accreditation (GEDA) accreditation [3].

The hypothesis of the study is that the provision of walking aids enhances mobility in living spaces and will have a significant impact on fear of falling, functionality, and quality of life. Therefore, this study aims to evaluate the efficacy of a program of training and provision of walking aids, associated or not with telemonitoring, on fear falling, mobility, quality of life, and risk of falls up to 3 and 6 months in older adults cared for in an ED. In addition, we hypothesize that fear of falling may be correlated with lower scores in mobility, quality of life, and risk of falls.

## Materials and methods

### Study design

**Fig 1:**
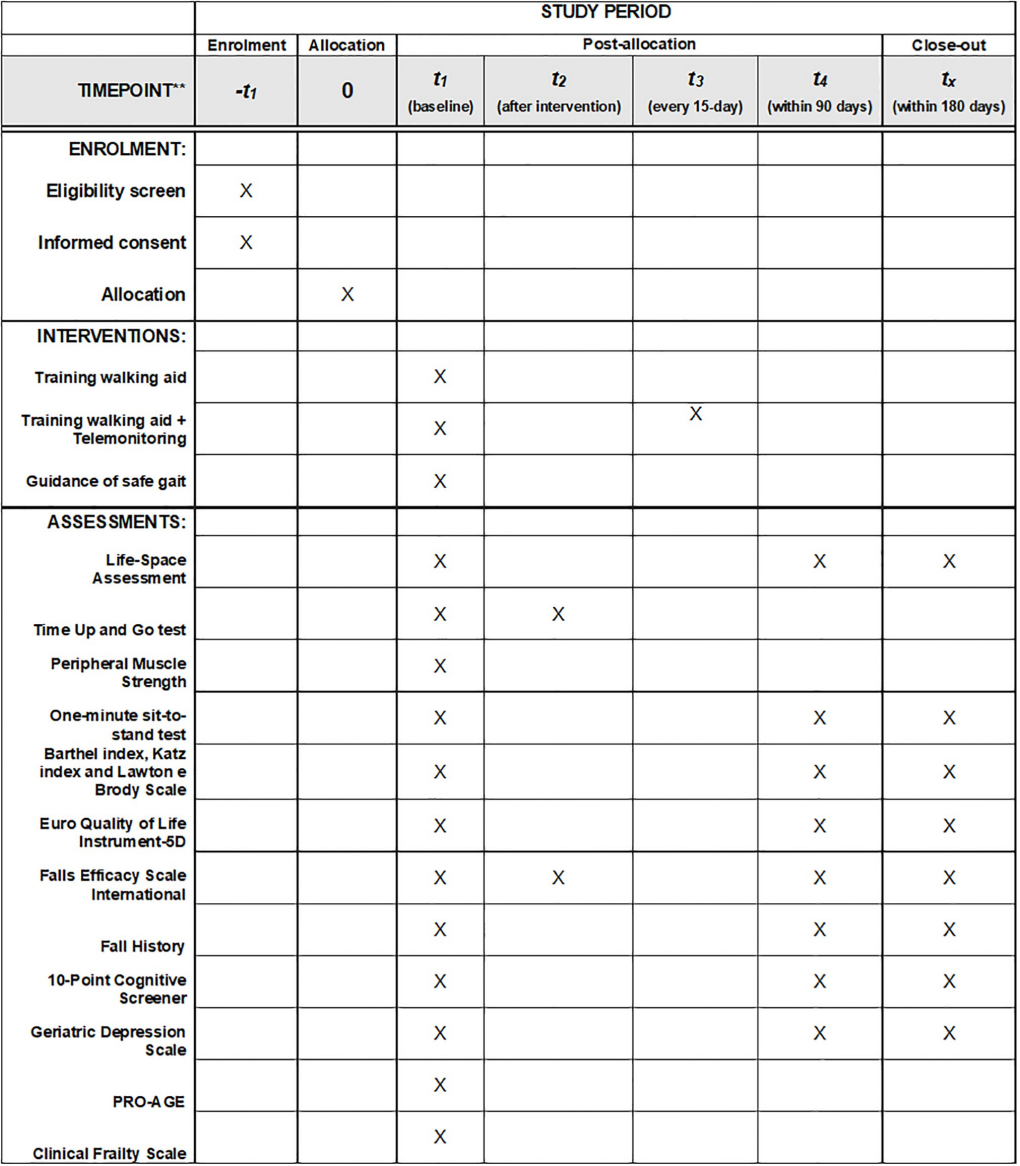
SPIRIT diagram.

**Fig 2:**
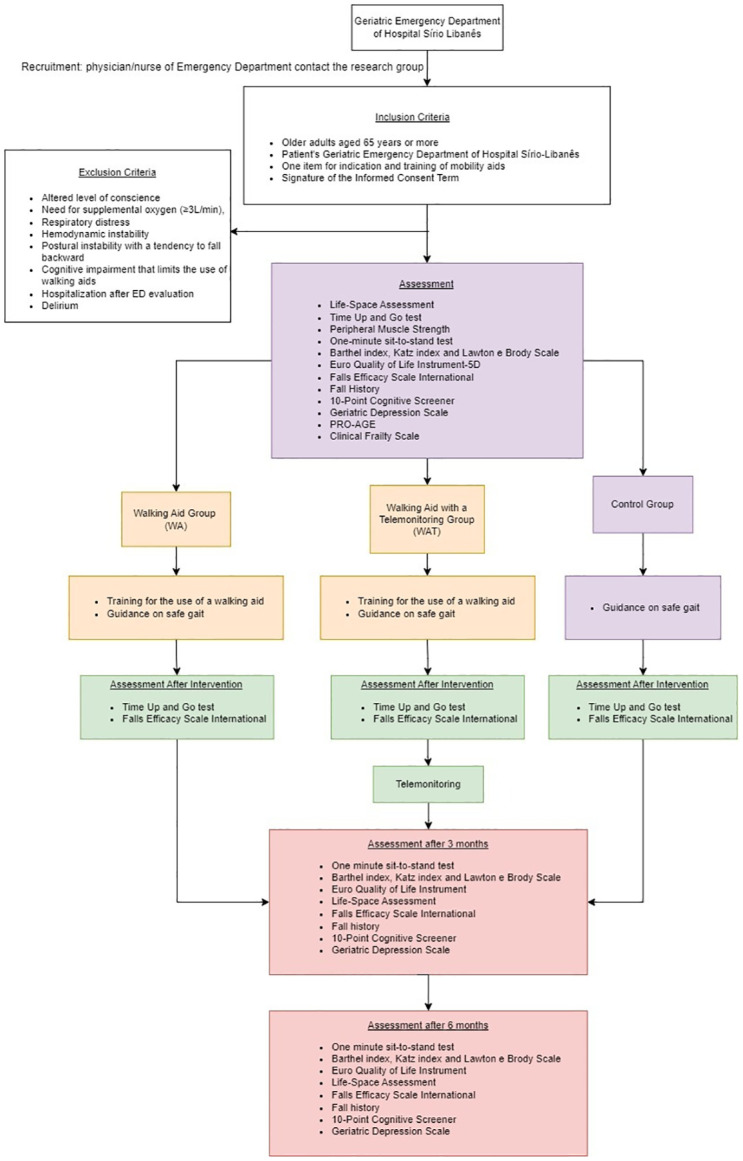
Flow diagram of the clinical trial.

The present study is a randomized single-blind controlled trial, and it was approved by the Ethics Committee of the Hospital Sírio-Libanês (number 6.430.440), and the protocol design was registered in the Clinical Trials database (NCT05950269). This protocol was structured according to Standard Protocol Items: Recommendations for Interventional Trials (SPIRIT) (S1 File). As this is a study protocol, no data have been included and conforms to PLOS data policy. The overall schedule and time commitment for trial participants are depicted in Fig 1. The patients will be recruited by convenience from the geriatric ED of a philanthropic and tertiary hospital in São Paulo, Brazil, according to inclusion and exclusion criteria. The research structure is shown in Fig 2.

### Sample

Sample size calculation was based on Morgan and cols study, which described a minimal detectable change (MDC_95_) of 8,2 points on Falls Efficacy Scale International (FES-I), with a standard deviation of 12 points [11]. For the calculation, ANOVA sample size was used, and three groups were considered. For an α of 0.05 and a power of 0.80, the total sample size was computed as 42 individuals. Due to the estimated loss of 20% during follow-up, the sample necessary for the success of the study was expanded to 153 individuals, 51 in each group (randomization 1:1:1).

We will include patients aged 65 years or older, admitted to the Geriatric ED of Hospital Sírio-Libanês, willing and able to give informed consent and with at least one of the following for indication and training of mobility aids: reduction of postural instability; improvement of motor control; increase of somatosensory feedback; reduction of biomechanical overload; safe promotion of autonomy; fall history (in the last six months). Exclusion criteria will include altered level of conscience, need for supplemental oxygen (≥3L/min), respiratory distress, hemodynamic instability, postural instability with a tendency to fall backward, cognitive impairment that limits the use of walking aids, hospitalization after ED evaluation, and delirium.

### Data handling

Data used in this study will be collected and managed using the software *“Research Electronic Data Capture”* (REDCap) [12] hosted on the servers of the Hospital Sírio-Libanês. REDCap is a safe software web-based and designed to capture information, providing: 1) an intuitive interface for validated data collection; 2) audit trails for screening data manipulation and export procedures; 3) automated export procedures for continuous data downloads to common statistical packages; 4) procedures for importing data from external sources [12]. REDCap is a secure, browser-based web application widely used by researchers for survey data collection. REDCap offers unique features that can be used to conduct rigorous randomized controlled trials (RCTs).

Research assistants will have a tablet with online access to the study database through login and password to fill out the screening, application, and signature of the Informed Consent Term through the functionality of REDCap eConsent framework, and complete the assessment data during the project.

Data to be collected at baseline include age, sex, social and demographic parameters, clinical parameters, detailed medication history, functionality, cognition, quality of life, mobility in life spaces, gait speed, muscle strength, fear of falling, and fall history.

### Randomization and blinding

The process of randomization will be carried out by the software REDCap (automatically, without contact with the researcher) after the baseline evaluation. Subjects will be allocated to one of the intervention groups (1:1:1), which will have the same number of participants each: walking aid (WA) group; walking aid with telemonitoring (WAT) group; control group. The WA group will be trained for the use of a walking aid and receive guidance on safe gait but will not receive telemonitoring. The WAT group will receive training for the use of a walking aid, guidance on safe gait, and telemonitoring. The control group will receive only guidance on safe gait.

This study will be blind for the researchers involved in the post intervention assessments and the researcher who will analyze the data. Also, the data will be analyzed by a researcher who is not involved in the interventions nor the assessments.

### Procedures

#### Recruitment

The recruitment will be carried out by a researcher in the Geriatric ED of the Hospital Sírio-Libanês (São Paulo, Brazil), who will actively search for eligible subjects or will be called by a physician or a nurse on duty.

#### Screening

The volunteer participants will undergo a screening process to ensure the eligibility criteria for this study. In this step, the participants are going to be interviewed for data collection (social, demographic, clinical, and medication history). One of the exclusion criteria is delirium, which is going to be evaluated by the Confusion Assessment Method (CAM), a fast instrument that can be used by clinical physicians and researchers without the need for a psychiatric qualification [13].

#### Assessment moments

Baseline assessment will be carried out before randomization and interventions. Assessment of gait speed and fear of falling will be repeated immediately after the intervention. Fear of falling, life-space mobility, functionality, quality of life, fall history, cognition and mood assessments will be repeated 3 and 6 months after intervention by a telephone interview or video call.

#### Mobility in life-space

Mobility in life space will be a secondary outcome of the study and will be assessed by the LSA, which allows the characterization of mobility in life-spaces (other rooms of your home besides the room where you sleep; to an area outside your home, places outside your neighborhood but within your town; and to places outside your town), specifically frequency, need for mobility aids and the help of third party in the last 4 weeks [14]. Four indicators are calculated (with the highest scores indicating greater life space):

1. Independent life-space: the highest life-space level attained without help from a person or mobility aid.
2. Life-space using equipment: the highest life-space level attained when a mobility aid was used, without help from a person.
3. Maximal life-space: the highest life-space level attained with the help of a person or mobility aid.
4. Composite score: encompasses distance, frequency, and level of independence, with a range from 0 to 120.

#### Gait assessment

Timed Up and Go test (TUG) evaluate mobility, balance, gait, and risk of falling [15]. In TUG, the researcher requests the participant to stand up on the chair (without using arms), walk 3 meters, go around outside a cone, walk back to the chair, and sit down without using arms [16]. Patients will be instructed to perform the test at the highest possible speed three times and the average of them will be used as the result [16].

#### Peripheral muscle strength

Peripheral muscle strength will be measured by handgrip strength (HGS) with a hand-held dynamometer (model SH 5001, brand SAEHAN), respecting the protocol recommended by the American Association of Hand Therapists (ASHT). Participants are going to be guided to remain seated, with the shoulder in a neutral position, one hand resting on the thigh and the elbow of the member that will be measured must remain flexed at 90 degrees, with the forearm in neutral rotation [17]. The interval between attempts will be one minute. The dominant hand will be used and the best score between the three measures is going to be considered a handgrip strength measure.

#### Functional capacity

Functional capacity is defined as the ability to perform activities of daily living (ADL) with autonomy [18, 19]. In this study, it will be evaluated with the one-minute sit-to-stand test, the Barthel index, the Katz index, and the Lawton-Brody scale.

One-minute sit-to-stand test will be based on a protocol described by Ozalevli and cols [20]. With the hands on the hip, participants will be guided to sit and stand completely in a chair (height 46 centimeters) as often as possible during 1 minute.

Katz index will be used to evaluate basic ADL. This index covers self-care, including feeding, bathing, dressing, toileting, transferring, and continence, with scores ranging from 0 (independent) and 1 (dependent) for each item [21, 22]. As a result, participants will be classified from 0 (independency for ADL) to 6 (total dependency for ADL) [21, 22].

The Barthel index evaluates the autonomy for self-care, in addition to mobility [23–25]. It is a tool that covers 10 tasks: feeding, bathing, grooming, dressing, bowels, bladder, toilet use, transfers (bed to chair and back), mobility (on level surfaces), and stairs [23–25]. The final score ranges from 0 to 100 and each item is scored according to how the individual performs each task (independently, with some help, or dependently). The higher the score, the higher the independency: 80 to 100 indicate independence; 60–79 points for slight dependency; 40–59 points for moderate independence; 20–39 points for severe dependency; 0–20 points for total dependency [23–25].

Instrumental ADL will be evaluated by the Lawton-Brody scale, which includes seven activities: the ability to use the telephone, mode of transportation, shopping, housekeeping, food preparation, responsibility for own medications, and ability to handle finances [26]. Each item has three possible answers: independent (3 points), need help (2 points), and unable to perform the task (1 point) [26]. The final score classifies the individuals as independent (21 points), partially dependent (8–21 points), or totally dependent (7 points) [27].

#### Quality of life

Quality of life will be evaluated by Euro Quality of Life Instrument-5D (EQ-5D) [28]. This instrument covers five health dimensions: mobility, self-care, usual activities, pain/discomfort, and anxiety/depression [29]. Each dimension is classified into 3 levels: no problems (1 point), some problems (2 points), and extreme problems (3 points) [30]. The results will be presented as frequency distributions of levels and dimensions over time, including changes within dimensions [29].

#### Fear of falling

Fear of falling will be evaluated by Falls Efficacy Scale International (FES-I) [31]. This tool has 16 items that evaluate, for example, walking on a slippery surface or an uneven surface, visiting a friend/relative, or attending a social event [32]. The fear of falling in each activity is classified on a 4-point scale (1—not at all concerned to 4—very concerned) [32]. The total score ranges from 16 to 64: 16–22 (low concern), 20–27 (moderate concern), and 28–64 (high concern) [32].

#### Fall history

Fall is a sudden and involuntary transfer of the body to the ground or a lower level [33]. Fall history will be evaluated periodically during the study and the patients must fill out a diary to register every moment in which they fall, and at the end of the study, this data (including location, associated injuries, need for special care after the fall) and the total number of falls will be evaluated.

#### Cognition and mood assessment

Cognitive assessment will be performed using the 10-Point Cognitive Screener (10-CS), a tool that consists of a brief cognitive screening, easily applicable and with greater accuracy and advantages [34]. It covers an evaluation of temporal orientation (1 point), verbal fluency (0 to 4 points), and three-word recall (1 point for each word) [34]. In the end, the score is adjusted for the education degree, adding 2 points for illiterate and 1 point for individuals with 1 to 3 years of education. Results are interpreted as follows: ≥ 8 points–normality, 6 to 7 points—possible cognitive impairment (usually mild cognitive impairment), and, 0 to 5 points—probable cognitive impairment (usually dementia) [34].

Assessment of mood disorders will be evaluated by the Geriatric Depression Scale (GDS-15) [35], which consists of 15 items (possible answers Yes or No) and a score of 0 or 1. The final score greater or equal to 5 points indicates the presence of significant symptoms of depression [35].

#### Geriatric vulnerability and frailty assessment

Geriatric vulnerability will be evaluated by the PRO-AGE scoring system, a fast tool developed to assess older patients in the ED [36]. Variables include functional impairment, recent hospitalization, advanced age, acute mental change, weight loss, male sex and fatigue.

Frailty will be evaluated using the Clinical Frailty Scale (CFS), which summarises the level of fitness or frailty of an older adult after evaluation by a health care professional [37]. Patients will be classified in one of the following categories: 1- very fit; 2- well; 3- managing well; 4- vulnerable; 5- mildly frail; 6- moderately frail; 7- severely frail; 8- very severely frail; 9- terminally ill [38].

#### Medication assessment

Medications used by patients will be tabulated with a detailed description including active ingredients, presentation, dose, and frequency. They will also be classified according to Beers criteria for potentially inappropriate medication use in older adults [39]. This criterion is categorized as 1) medications considered as potentially inappropriate; 2) medications potentially inappropriate in patients with certain diseases or syndromes; 3) medications to be used with caution; 4) potentially inappropriate drug–drug interactions; 5) Medications whose dosages should be adjusted based on renal function [39].

### Interventions

The interventions will be performed according to the randomization after the baseline data collection.

#### Training of walking aids

Training will be carried out with patients from groups WA and WAT. A physical therapist will identify the mobility needs and will indicate the most appropriate walking aid (cane or walker) considering the following parameters.

- Canes: one upper limb used for a walk, light weight-bearing and need for somatosensorial feedback; the cane must be positioned between 15 and 20 centimeters laterally to the feet; the patient’s hand should be supported on the cane at the height of the greater trochanter of the femur and the elbow should be flexed approximately by 30°; in general, the cane is used on the opposite side of the injured leg.
- Walkers: both upper limbs used to walk, heavy weight bearing, and presence of postural instability; the equipment must be held between 20 and 25 centimeters in front of the body with relaxed shoulders, erect torso, and elbow flexed at 20° to 30°; during the total weight-bearing gait, the walker should be lifted and move forward approximately arm’s length, while one lower limb move forward followed by the other, and the cycle repeats; in a non-weight bearing gait, the walker is lifted and moves forward, then the weight is transferred to the walker through the upper limbs, the affected limb is held in a position anterior to the person’s body, but does not contact the ground, the unaffected limb is moved forward and this cycle repeats.

#### Telemonitoring

Follow-up will be carried out only in the WAT group. Telemonitoring will occur every two weeks for three months after the ED discharge, through video call (about 15 minutes). On these opportunities, the importance of using mobile devices and the guidance on safe gait will be reinforced.

#### Guidance on safe gait

Guidance on safe gait will be carried out for all the groups in the study. Subjects will be instructed on strategies for fall prevention [40] and will receive a printed leaflet summarizing it (S2 File).

### Outcomes

#### Primary outcome

The primary outcome will be fear of falling.

#### Secondary outcomes

Secondary outcomes will include mobility, functionality, quality of life, mood, cognition, and occurrence of falls in three months.

### Statistical analysis

Continuous variables will be expressed on average and standard deviation or median and interquartile interval 25%-75%. Categorical data will be presented in absolute and relative numbers. To verify the distribution normality of data, the Shapiro-Wilk normality test will be applied. For comparisons between groups over time, One-way ANOVA test for parametric data or Kruskall-Wallis for non-parametric data will be used. For intragroup analyses, repeated measures Analysis of Variance (RM-ANOVA) test for parametric data or Friedman test for non-parametric data will be used. Only for the Time Up and Go variable before and after intervention, a paired t-test will be used for parametric data, or a Wilcoxon test will be used for non-parametric data. For intergroup and intragroup analyses, the baseline, 3 and 6 months after discharge from the geriatric ED will be considered. To evaluate correlations, the Pearson correlation test will be used for parametric data and the Spearman test will be used for non-parametric data. All the analyses will be carried out using the Statistical Package for Social Sciences (SPSS) version 28.0.1 (SPSS Inc.^®^; Chicago, IL, USA), considering a significance level of 5%.

## Discussion

International guidelines for Geriatric ED exist to guide and promote best practices for older patients [3, 4]. The American College of Emergency Physicians created an accreditation for geriatric EDs that abide by their recommendations and Hospital Sírio-Libanês was awarded a Level 3 (Bronze) for the first time in 2019, being the first hospital in the South Hemisphere with this recognition [3]. Although the provision of walking aids in the Geriatric ED is recommended in American and European guidelines [3, 4], it is based mainly on expert opinion and there is a lack of trial data on the impacts of training and provision of walking aids in this acute setting. Therefore, this study aims to be the first randomized controlled trial to evaluate this intervention, associated or not with telemonitoring.

Older adults have changes inherent to age, such as sarcopenia, decreased strength and muscle mass, and balance and gait disorders, which predispose to a greater risk of falls [41, 42]. Different than other studies carried out in Geriatric ED, the primary outcome will be the older patient’s fear of falling and the secondary outcomes will focus more on mobility than on falls and its consequences. With this strategy, this study aims to evaluate the impact of the walking aid on fear of falling, mobility and autonomy of older adults admitted to a Geriatric ED.

Previous studies carried out with older adults in the ED that explored fear of falling were mainly observational and aimed at its impact on risk and number of falls, ADL and quality of life [43–45]. On the other hand, experimental studies in older patients that assessed fear of falling were carried out exclusively in other environments, such as ambulatory settings [46]. Finally, Goldberg et al. carried out a randomized controlled trial aimed to determine if a fall prevention program in the ED could reduce revisits and hospitalizations related or not to falls, but, despite including in this program a physical therapist assessment, there was no training and provision of walking aids [47].

Telemonitoring has been more frequently studied in older adults, but mainly in ambulatory settings and focused on chronic illnesses [48–50]. In this study, the intervention will be evaluated in acutely ill older patients admitted to a Geriatric ED and trained in the use of walking aids. In addition, in a randomized clinical trial, telemonitoring could be an additional strategy to reduce the loss of follow-up of participants during the study.

Finally, to the best of our knowledge, there are no previous studies that have compared groups according to the provision of gait assistance devices and used FES-I as a main outcomes. Therefore, we’ve calculated the sample size using as a reference the study by Morgan et al. [11] which proposed a gait assistance device intervention in its participants and had a higher reported DMC than other initiative [51] but conducted intragroup comparisons. In addition, our study will also address intragroup differences when comparing baseline, 3 and 6 months after discharge from the geriatric ED.

A limitation of the study protocol is that it will be performed in a single center. However, having been described in detail, registered in a public database, and published in an open-source journal, it might be replicated by other research groups around the globe. In addition, another limitation of the study is that participants in the control group may initiate the use of assistive gait devices over the 6-month study period. However, this identification will be possible through the LSA questionnaire. After completion of the study and blind analysis of the data by the statistician, the percentage of individuals in the control group who initiated the use of gait devices will be shown. If there are patients in the control group who initiated the use of a gait device, it will be considered a protocol deviation from the assessment point at which they started using the assistive gait device, which may be 3 or 6 months after discharge from the geriatric ED service. Consequently, a secondary analysis will be presented without the protocol deviation values.

## Supporting information

S1 File Standard protocol items: Recommendations for interventional trials (SPIRIT). SPIRIT Checklist: Recommended items to address in a clinical trial protocol and related documents.
(DOC)

S2 File Guidance for a safe gait. (DOCX)

S3 File (PDF)

S4 File (PDF)

S5 File (PDF)
