# Supplementary material for: Training and provision of mobility aids to promote autonomy and mobility of older patients in a geriatric emergency department: A protocol for a randomized controlled trial
Source: PLoS One. 2024 Jul 31;19(7):e0304397. doi: 10.1371/journal.pone.0304397 (PMC11290684; doi:10.1371/journal.pone.0304397)
Supplement: S2 File — (DOCX) [file pone.0304397.s002.docx]

**S2 File**

***Guidance for a Safe Gait***

1. Stand up

- To get out of bed, first roll the body, flex the knees and put the legs out of the bed. Stand up with the support of arms and support tightly the feet on the floor. Wait a few minutes to prevent dizziness and imbalance.
- To stand up, support tightly both feet on the floor, slightly apart. Lean the torso forward. If necessary, use the support of your arms, the walking aid (cane or walker), or someone’s help.
- Observe the stability on standing position; only if you stay steady with legs, torso and hip, without dizziness, you can start walk.

1. Walk

- Use supports, such as walking aids (cane or walker), another person or bar if necessary. Talk with your physician and/or physical therapist about walking aid indication to give you more safety and independence to walk.
- Try to walk slowly to better control your steps.
- Prefer comfortable and safe shoes that stick to the foot, avoiding slippers, very wide shoes, socks, high heels and shoes with slippery sole.
- Wear glasses and hearing aid if necessary. Keep glasses and/or hearing aids well adapted (regularly visit the ophthalmologist and/or otorhinolaryngologist).
- Avoid leaving objects in circulations areas that could cause an accident, such as rug and exposed wires, because of the risk of falling. Always keep the environment well lit.
- Walking time: according to medical advice. If you couldn’t walk long distances, walk short distances more times a day.
- Always observe signs of tiredness (breathless, muscle or bone pain, lower limb tremors, intense sweating and excessive increase in heart rate) or imbalance. If you present one or more of these signs, interrupt the walk and rest. If the signs persist, contact your physician.

1. Go up and down the stairs

- Always use the stairs’ handrails.
- Avoid narrow steps.
- Go up and down one step at a time.
- To go up the stairs, start by putting the foot of the strongest leg on the step and then the other foot, supporting both feet on the same step.
- To go down the stairs, start by putting the weakest leg on the step and then the other leg on the same step, going down one step at a time.
