# Supplementary material for: Training and provision of mobility aids to promote autonomy and mobility of older patients in a geriatric emergency department: A protocol for a randomized controlled trial
Source: PLoS One. 2024 Jul 31;19(7):e0304397. doi: 10.1371/journal.pone.0304397 (PMC11290684; doi:10.1371/journal.pone.0304397)
Supplement: S3 File — (PDF) [file pone.0304397.s003.pdf]

PARECER CONSUBSTANCIADO DO CEP

DADOS DA EMENDA

Título da Pesquisa: Treinamento e fornecimento de dispositivos auxiliares de marcha para promoção de autonomia e mobilidade de idosos em pronto atendimento: um ensaio clínico randomizado.

Pesquisador: Pedro Kallas Curiati

Área Temática:

Versão: 3

CAAE: 67316323.9.0000.5461

Instituição Proponente: Sociedade Beneficente de Senhoras Hospital Sírio-Libanês

Patrocinador Principal: Sociedade Beneficente de Senhoras Hospital Sírio-Libanês

DADOS DO PARECER

Número do Parecer: 6.430.440

Apresentação do Projeto:

As informações elencadas nos campos "Apresentação do Projeto", "Objetivo da Pesquisa" e "Avaliação dos Riscos e Benefícios" foram retiradas do arquivo Informações Básicas da Pesquisa (PB_INFORMAÇÕES_BÁSICAS_2219507_E1.pdf de 26/09/2023).

Desenho:

Ensaio clínico randomizado.

Resumo:

INTRODUÇÃO: Idosos apresentam maior taxa de admissão no pronto atendimento (PA) quando comparados a população jovem. Mobilidade é a capacidade física de uma pessoa se locomover ou movimentar, mas também abrange o ambiente da pessoa e a capacidade de se adaptar ao mesmo. A redução de mobilidade resulta em diminuição de convívio social, isolamento e solidão, afetando aspectos físicos, psicológicos e sociais do idoso. Dispositivos auxiliares de marcha podem ser utilizados para melhorar a mobilidade e prevenir quedas. Segundo diretrizes internacionais, devem estar disponíveis no ambiente de PA. OBJETIVOS: Avaliar a eficácia de um programa de treinamento e fornecimento de dispositivos auxiliares de marcha, associado ou não a telemonitoramento, em mobilidade e equilíbrio funcional, assim como velocidade de marcha, qualidade de vida, medo de

cair e número de queda em 3 meses, em idosos atendidos em serviço de PA. MÉTODOS: Será realizado um ensaio clínico randomizado em que serão recrutados indivíduos idosos em atendimento no PA do Hospital Sírio Libanês (HSL). Os critérios de inclusão serão: idade superior ou igual a 65 anos; atendimento no PA do HSL; ao menos 1 indicação para uso de dispositivos auxiliares da marcha; e assinatura do Termo de Consentimento Livre e Esclarecido (TCLE). Os critérios de exclusão incluem: alteração do nível de consciência; necessidade de oxigênio suplementar (3L/min); desconforto respiratório; instabilidade hemodinâmica; instabilidade postural; comprometimento cognitivo que dificulte o uso de dispositivo auxiliar de marcha; internação após avaliação inicial no serviço de PA; delirium. Os participantes serão randomizados e alocados em três grupos de intervenção, sendo: A) Grupo dispositivo auxiliar de marcha (GDAM), em que os pacientes receberão treinamento com dispositivo auxiliar de marcha e orientações sobre marcha segura, mas não receberão telemonitoramento; B) Grupo dispositivo auxiliar de marcha e telemonitoramento (GDAM+Tele), em que os pacientes receberão treinamento com o dispositivo auxiliar de marcha, orientações sobre marcha segura e telemonitoramento; C) Grupo controle (GC), em que os pacientes receberão somente orientações sobre marcha segura e não receberão nem treinamento com o dispositivo auxiliar de marcha nem telemonitoramento. Os pacientes serão avaliados quanto a idade, sexo, dados sociais e demográficos, dados clínicos, mobilidade nos espaços de vida, velocidade da marcha, força muscular, funcionalidade, qualidade de vida, medo de cair, histórico de quedas, e avaliação cognitiva e psicossocial. Toda a avaliação basal será realizada antes da intervenção. A avaliação de tempo da marcha e medo de cair será repetida após a intervenção. Além disso, as avaliações de funcionalidade, qualidade de vida, medo de cair, histórico de quedas, cognição e estado psicossocial serão repetidas após 3 meses da alta do pronto atendimento geriátrico através de entrevista telefônica. Será considerado como desfecho primário a mobilidade nos espaços de vida e como desfechos secundários velocidade de marcha, funcionalidade, força muscular, qualidade de vida, preocupação com a queda e ocorrência de queda em 3 meses. Para análise estatística, serão utilizados teste t pareado, teste de Wilcoxon, One-Way ANOVA, Kruskall-Wallis, correlação de Pearson e teste de Spearman de acordo com a normalidade estabelecida pelo teste de normalidade Shapiro Wilk. Será considerado significante quando $P<0,05$.

Introdução:

Segundo dados da Organização das Nações Unidas (ONU) e da Organização Mundial da Saúde (OMS), o Brasil e o mundo têm experimentado um aumento pronunciado de sua população idosa

nas últimas duas décadas (1). Com o objetivo de compreender melhor o impacto do envelhecimento populacional na sociedade, no meio ambiente e na economia global, a ONU, por meio de seu Departamento de Economia e Questões Sociais (Department of Economics and Social Affairs, DESA), realizou um estudo que identificou o Brasil e o continente sul-americano como expoentes dessa transição demográfica (2). Por meio de gráficos, o DESA descreveu o aumento da fração de idosos na população brasileira, que representava menos de 5% em 1990, atualmente representa algo entre 7% e 9% e estima-se que em 2050 representará mais de 15% (2). Nesse contexto, o serviço de pronto atendimento (PA) presta um papel relevante para a população idosa, constituindo um centro de tratamento de emergências, um ponto de entrada para o cuidado agudo de alta complexidade e para o acesso a serviços continuados de atenção à saúde, uma fonte de cuidado médico acessível 24 horas por dia, e uma rede de segurança quando a transição suave do cuidado entre sistemas de atenção à saúde é comprometida (3, 4). De acordo com o Centro Americano de Estatísticas de Saúde (National Center for Health Statistics), departamento do Centro de Controle e Prevenção de Doenças (Centers for Disease Control and Prevention, CDC) dos Estados Unidos, em 2018 pessoas com mais de 74 anos representavam 56,7% da demanda anual em serviços de PA (5). Entre 2002 e 2012, na Suíça, os atendimentos de urgência de idosos, com 60 anos de idade ou mais, cresceu 42,3% em comparação a apenas 27,6% em faixas etárias mais jovens (6).virtude de seus múltiplos e interrelacionados problemas médicos e sociais, idosos geralmente desafiam os sistemas de cuidado tipicamente desenvolvidos para atender indivíduos com distúrbios de um único aparelho e com redes de suporte social robustas (7) (8). Quando comparados com a população mais jovem, pacientes geriátricos são mais frequentemente admitidos nos serviços de PA trazidos por Em ambulância, triados como urgência ou emergência e encaminhados para internação hospitalar (9) (10). De fato, em 2015, nos Estados Unidos, 29% das visitas de idosos com idade superior ou igual a 75 anos culminou em internação hospitalar e estadia média de 5 dias, enquanto apenas 5% das visitas de adultos com idade de 25 a 44 anos tiveram o mesmo desfecho (9). Nos Estados Unidos, em 2018, 16,1% dos atendimentos de idosos nos serviços de PA foram classificados como emergência, 40,2% foram classificados como urgência e 17,7% culminaram em internação (11). Apesar de idosos serem submetidos a maior número de exames e procedimentos do que pacientes mais jovens, os seus diagnósticos tendem a ser menos precisos, o que pode ser atribuído a uma maior frequência de apresentações atípicas de doença, polifarmácia e multimorbidades (3, 7-10, 12-19). O resultado é que médicos emergencistas referem maior dificuldade no manejo de pacientes idosos (3, 20), e muitos idosos se queixam de falta de resolutividade dos serviços de PA quanto as suas queixas (3, 21, 22). Além disso, visitas a

serviços de PA nessa população estão associadas a altas taxas de internação em unidades de cuidados agudos, internação hospitalar prolongada, readmissão hospitalar não-programada, dependência funcional e mortalidade (7, 10, 15, 23-26). No entanto, seria muito simplista atribuir o pior prognóstico de idosos estritamente a fatores clínicos, sendo necessário considerar que os sistemas de cuidado à saúde possivelmente estão menos preparados para lhes atender (10, 22). Como estratégias para o cuidado diferenciado aos idosos pode-se incluir programas com modelos de avaliação geriátrica ampla, equipes de transição de cuidado, protocolos de alta facilitada, atendimentos em hospital dia, atenção domiciliar e internações em unidades de cuidados agudos geriátricos (10, 27).A avaliação geriátrica ampla (AGA) é um processo de diagnóstico interdisciplinar e multidimensional que visa determinar a situação clínica, funcional e psicológica do idoso frágil, a fim desenvolver um plano de tratamento e acompanhamento a longo prazo (27, 28). Como benefícios da implementação da AGA são descritos a melhora do desempenho cognitivo, menor perda funcional, menor taxa de institucionalização e mortalidade, e redução de internações desnecessárias e duração do tempo de internação hospitalar (10, 29-33). De acordo com as diretrizes internacionais, o atendimento do idoso no PA demanda políticas, protocolos e fluxos diferenciados, projetados para as particularidades dessa população (12). A diretriz europeia cita como principais recomendações para este público os “5 Ms da geriatria”: mente, medicamentos, multicomplexidade, “mais importante” e mobilidade. Em outras palavras, durante o atendimento do idoso no serviço PA, recomenda-se: abordagem de demência, delirium, depressão e comprometimento cognitivo; avaliação estruturada e revisão dos medicamentos que são utilizados pelos idosos, considerando possíveis interações medicamentosas e uso inapropriado de medicamentos; ponderação das necessidades gerais do idoso, sejam elas médicas, psicológicas, sociais, funcionais ou ambientais; quanto ao item “mais importante” recomenda-se garantir os resultados de saúde de maneira individualizada focando nos objetivos que tenham significado para o idoso (34). Recomenda-se também que o espaço físico do PA geriátrico foque em modificações estruturais que visem a segurança, conforto, pistas de memória e percepção sensorial (tanto visão quanto audição), garantindo maior mobilidade dos idosos. Além disso, as diretrizes destacam que as melhorias para mobilidade e segurança, estão relacionados não apenas ao mobiliário, mas também reforçam a importância do fácil acesso a dispositivos auxiliares de marcha (12). A mobilidade é um termo utilizado não apenas para descrever a capacidade física de uma pessoa se locomover ou movimentar, mas também abrange o ambiente da pessoa e a capacidade de se adaptar ao mesmo (35). Apresenta relação direta com a funcionalidade, independência, autonomia e qualidade de vida do indivíduo (35) e pode ser avaliada tanto pela

capacidade do indivíduo se transferir de um mobiliário (cama ou cadeira), caminhar e subir escadas, como também pelo espaço de vida, que é definido pela distância que ele consegue percorrer dentro e fora do domicílio, com ou sem assistência (35). As limitações de mobilidade são comuns nos idosos e estão associadas a sintomas depressivos e diminuição da qualidade de vida, visto culminam em diminuição de convívio social, isolamento e solidão, afetando aspectos físicos, psicológicos e sociais do idoso (36). Os fatores de risco que estão mais associados à limitação de mobilidade são idade avançada, baixa atividade física, obesidade, alteração de força e equilíbrio, alterações de marcha e doenças crônicas, além de outros fatores menos relatados como sintomas depressivos e déficit cognitivo, ingestão de álcool, tabagismo e hospitalização recente (35). Portanto, a avalição de mobilidade deve ser considerada um componente a ser incluído no cuidado de saúde do idoso (35).Correlacionado com a mobilidade, atualmente utiliza-se o termo de espaço de vida, um conceito emergente relacionado a fatores funcionais, ambientais e sociais que definem como as pessoas experimentam seu cotidiano (37). Uma revisão sistemática publicada em 2019 revelou que a mobilidade no espaço de vida apresenta associação moderada com função cognitiva, já que prevê execução de ação, aprendizagem, memória, atenção, habilidade visuoespacial e função percepto-motora. Estes fatores de função cognitiva estão ligados à autonomia do idoso, e tanto a mobilidade quanto a função cognitiva podem apresentar declínio durante o envelhecimento (38). Nesse contexto, a University of Alabama at Birmingham desenvolveu em 2003 um instrumento de baixo custo, que não necessita de espaço, equipamentos ou treinamentos específicos, e que pode ser aplicado de maneira presencial ou através de contato telefônico: o Life Space Assessment (LSA) (39). Este instrumento foi validado em um estudo brasileiro em 2018, que avaliou 80 idosos com idade acima de 60 anos recrutados em serviços públicos de saúde (ambulatório e atendimento domiciliar) (39). Concluiu-se que o LSA apresenta confiabilidade, validade e reprodutibilidade adequadas para avaliação da mobilidade nos espaços de vida de idosos brasileiros, com de Cronbach=0,92 e ICC = 0,97, semelhante à versão original (ICC = 0,96) (39). Avalia a mobilidade do idoso no espaço de vida, em cinco cenários diferentes, nas quatro semanas anteriores, com descrição de frequência e independência para cada nível (39). As subpontuações de mobilidade no espaço de vida são obtidas para cada nível de espaço de vida (1-5) multiplicando frequência e independência. Posteriormente, são somadas as subpontuações de cada nível para obter a pontuação final da LSA que varia de 0 a 120, em que 0 refere-se a um indivíduo restrito ao cômodo onde dorme e 120 para o indivíduo que se locomove diariamente de maneira independente para outras cidades (40, 41). Um estudo demonstrou que a nota de corte para risco de declínio nas atividades instrumentais de vida diária seria de 56 pontos (42). A LSA é

uma ferramenta que reconhece que fatores cognitivos e funcionais afetam a mobilidade, a qualidade de marcha (ritmo, velocidade, variabilidade da marcha) e a capacidade do indivíduo realizar suas atividades do dia a dia (43). Um estudo realizado em 2016, nos Estados Unidos, acompanhou durante 3 anos idosos acima de 75 anos através de uma entrevista inicial domiciliar e entrevistas telefônica mensais com o objetivo de avaliar a associação de visitas ao serviço de PA e necessidade de hospitalização com mobilidade comunitária (44). A mobilidade foi avaliada através da ferramenta LSA e foram registrados motivos das admissões, comorbidades dos pacientes e atividades de vida diária através do índice Katz (44). Observou-se que os idosos que necessitaram de uma visita ao serviço PA (cerca de 20% dos participantes) ou internação hospitalar (cerca de 40%) tiveram menor escore LSA, que não retornou ao valor prévio ao contato com o serviço de saúde mesmo após um ano (44).Dispositivos auxiliares de marcha promovem a independência dentro do ambiente através de estabilização biomecânica, melhora do equilíbrio e do controle motor, feedback sensorial, redução da carga dos membros inferiores, ganho de confiança e prevenção de quedas (45, 46). Em contrapartida, podem aumentar o risco de queda, tanto por uso inadequado como por indicação para pacientes com déficit de equilíbrio e alterações prévias de marcha (45). Por esta razão, é recomendado que seu uso seja prescrito por um profissional habilitado e seja precedido por orientações, treinamento e acompanhamento para otimização dos benefícios, redução dos riscos e maior adesão e confiança (45, 46). Diversos estudos apontam o telemonitoramento como uma ferramenta para o cuidado a saúde (47). Ele é definido como o uso de tecnologia da informação e telecomunicações para o cuidado a saúde à distância (48). Em uma revisão sistemática publicada em 2019, foi considerado viável e bem recebido para o cuidado da saúde do idoso e recomendado para a prática clínica por superar barreiras de distância e acesso a serviços de saúde(49). Além disso, um estudo realizado em 2021, com 60 idosos frágeis, comparou o cuidado desta população durante um ano, sendo um grupo através de telemonitoramento com equipe multiprofissional e o outro com o acompanhamento convencional, demonstrando melhor resultado do telemonitoramento quanto a humor, comportamento, atividades de vida diária e estado nutricional (50). A literatura atual sobre telemonitoramento na população idosa e possíveis intervenções de fisioterapia abrange telemonitoramento e telereabilitação de idosos com doença pulmonar obstrutiva crônica (DPOC) e com doenças cardíacas, com evidências de eficácia para continuidade do tratamento domiciliar em programas de reabilitação após internações hospitalares (eventos agudos e pós-operatórios), promoção da reabilitação, melhora da capacidade funcional e redução de reinternações (51-53). Poucos estudos exploraram o telemonitoramento para a população no serviço de PA, apesar da evidência de que

essa intervenção em idosos com múltiplas doenças reduz o número de internações e visitas ao PA (54, 55). De fato, não há estudos que avaliem os efeitos de uma intervenção de dispositivos de auxílio a marcha, associado ou não ao telemonitoramento, n a mobilidade de idosos admitidos ao serviço de PA.Idosos agudamente enfermos que são admitidos por meio do PA com queixas associadas ou não às quedas têm um risco maior de quedas, com impacto em funcionalidade, qualidade de vida, cognição, revisitas e mortalidade (28). Diante disso, é fundamental que seja incorporado dentro de PA triagens e avaliações de mobilidade, qualidade da marcha e risco de queda em idosos e implementadas medidas para promoção de segurança, mobilidade e qualidade de vida deste paciente, o que inclui o uso adequado e seguro de dispositivos auxiliares de marcha. Cabe destacar que até o momento não há estudo que comprove sua segurança nem seu benefício no ambiente de cuidados agudos, a despeito das recomendações de diretrizes internacionais e da acreditação Geriatric Emergency Department Accreditation (GEDA) (12).

Hipótese:
Um programa de treinamento e fornecimento de dispositivos auxiliares de marcha, associado ou não a telemonitoramento, promove melhora de mobilidade e equilíbrio funcional em idosos atendidos em serviço de PA. Um programa de treinamento e fornecimento de dispositivos auxiliares de marcha, associado ou não a telemonitoramento, promove melhora em velocidade de marcha, qualidade de vida, medo de cair e número de queda em 3 meses em idosos atendidos em serviço de PA.

Metodologia Proposta:
- População
Serão recrutados indivíduos idosos que estarão em atendimento no PA do HSL.

Intervenções com igual número de pacientes, podendo ser:
1) Grupo dispositivo auxiliar de marcha (AM);
2) Grupo dispositivo auxiliar de marcha com telemonitoramento (GDAM+Tele);
3) Grupo Controle (GC).

- Grupos
• Grupo dispositivo auxiliar de marcha (GDAM): os pacientes receberão treinamento com o dispositivo de marcha e orientações sobre marcha segura, mas não receberão telemonitoramento;

• Grupo dispositivo auxiliar de marcha e telemonitoramento (GDAM+Tele): os pacientes receberão treinamento com o dispositivo de marcha, orientações sobre marcha segura e telemonitoramento;
• Grupo controle (GC): os pacientes receberão somente orientações sobre marcha segura e não receberão treinamento com o dispositivo de marcha nem telemonitoramento.

- Cegamento

O estudo será cego para o estatístico. Os dados serão analisados por um pesquisador que não participou das intervenções nem das avaliações. Além disso, o pesquisador que realizará a avaliação final do projeto também será cego. Os grupos serão codificados e não será possível identificar pertencimento a GDAM, GDAM+Tele ou GC.

Procedimentos

- Recrutamento

Os participantes serão recrutados no PA do HSL, Unidade Bela Vista, São Paulo, Brasil, por assistente de pesquisa treinado, que permanecerá de plantão por 25 horas semanais em turnos distribuídos entre 7:00 e 18:00 por até 6 meses. Este poderá ser acionado pela equipe médica e/ou de enfermagem, mas também fará busca ativa de potenciais candidatos para o estudo. Os pacientes elegíveis para a triagem do estudo serão contatados pela equipe de pesquisa para sua realização e para assinatura do Termo de Consentimento Livre e Esclarecido (TCLE).

- Triagem

Inicialmente, todos os participantes voluntários serão submetidos a um processo de triagem para assegurar o cumprimento dos critérios de elegibilidade neste estudo. Nesta etapa, os participantes serão submetidos a uma entrevista inicial para coleta de dados sociodemográficos, clínicos e de medicamentos. Além disso, outro fator a ser avaliado e que será considerado critério de exclusão é delirium, definido como síndrome cerebral orgânica caracterizada por distúrbio de consciência, atenção, percepção, pensamento, memória, comportamento psicomotor, emoção e ciclo sono-vigília (58, 59). Para esta avaliação será utilizado a Escala Confusion Assessment Method (CAM), ferramenta rápida que pode ser aplicada por clínicos e pesquisadores sem a necessidade de formação psiquiátrica (60). Esta ferramenta foi validada para a população de idosos nos serviços de PA, primeiramente no Canadá e posteriormente em um estudo brasileiro (61-63). Existem duas versões para sua utilização, a curta que apresentam 4 itens, utilizada para rastreio, e a versão longa, amplamente utilizada para confirmação diagnóstica, classificação e uso em pesquisa (60).

- Momentos avaliativos

Toda a avaliação basal será realizada antes da intervenção. A avaliação de velocidade da marcha e medo de cair será repetida após a intervenção. A avaliação de mobilidade nos espaços de vida, funcionalidade, qualidade de vida, medo de cair, número de quedas e cognição será repetida 3 meses após a intervenção por entrevista telefônica ou videochamada.

Critério de Inclusão:

Serão adotados os seguintes critérios de inclusão:

- Idade superior ou igual a 65 anos;
- Atendimento no PA do HSL;
- Preenchimento de pelo menos um critério do protocolo institucional para indicação e treinamento de dispositivos auxiliares da marcha no PA (Anexo 1): aumento da estabilidade postural; aumento do retorno somatossensorial; auxílio no controle motor; redução da sobrecarga biomecânica; promoção da autonomia com segurança; e histórico de quedas (seis últimos meses).
- Assinatura o Termo de Consentimento Livre e Esclarecido (TCLE).

Critério de Exclusão:

Serão considerados para exclusão do estudo os seguintes critérios:

- Alteração do nível de consciência;
- Necessidade de oxigênio suplementar (3L/min);
- Desconforto respiratório; o Instabilidade hemodinâmica;
- Instabilidade postural com tendência de queda para trás;
- Comprometimento cognitivo que prejudique manipulação do dispositivo auxiliar de marcha;
- Internação após avaliação no serviço de PA;
- Delirium

Metodologia de Análise de Dados:

As variáveis contínuas serão expressas em média e desvio padrão (DP) ou mediana e intervalo interquartil (IIQ) 25%-75%. Os dados categóricos serão apresentados em número absoluto e relativo (%). Com intuito de verificar a distribuição de normalidade dos dados, será aplicado o teste de normalidade Shapiro Wilk. Serão utilizados os testes t pareado para dados paramétricos ou o teste de Wilcoxon para dados não-paramétricos. Para avaliação entre os três grupos de

intervenção serão utilizados o teste One-Way ANOVA para dados paramétricos ou Kruskall-Wallis para dados nãoparamétricos. Para avaliar as correlações serão utilizados os testes de correlação de Pearson para dados paramétricos e o teste de Spearman para dados não-paramétricos. Todas as análises serão realizadas utilizando o pacote de estatístico software Statistical Package for Social Sciences (SPSS) versão 28.0.1 (SPSS Inc.®; Chicago, IL, USA), considerando nível de significância de 5%.

Desfecho Primário:
- Mobilidade nos espaços de vida.

Desfecho Secundário:
- Velocidade de marcha;
- Funcionalidade;
- Qualidade de vida;
- Preocupação com a queda;
- Ocorrência de queda em 3 meses.

Objetivo da Pesquisa:
Objetivo Primário:
Avaliar a eficácia de um programa de treinamento e fornecimento de dispositivos auxiliares de marcha, associado ou não a telemonitoramento, na mobilidade e no equilíbrio funcional de idosos atendidos em serviço de PA.

Objetivo Secundário:
Avaliar a eficácia de um programa de treinamento e fornecimento de dispositivos auxiliares de marcha, associado ou não a telemonitoramento, em velocidade de marcha, qualidade de vida, medo de cair e número de queda em 3 meses em idosos atendidos em serviço de PA.

Avaliação dos Riscos e Benefícios:
Riscos:
Os participantes voluntários deste estudo poderão relatar diferentes graus de desconforto, cansaço ou fadiga, principalmente durante a execução dos testes funcionais e de força e da

aplicação da entrevista. No entanto, estes sintomas deverão cessar após 5 minutos de repouso. Todas as informações coletadas serão compiladas e gerenciadas por meio do sistema de captura de dados eletrônicos (REDCap) hospedada nos servidores do Hospital Sírio-Libanês. Os dados serão disponibilizados aos pesquisadores de forma "de-identificada" com intuito de proteger a identidade do participante e garantir a integridade dos dados. O acesso à base de dados será limitado apenas aos investigadores membros da equipe de pesquisa. Existe o risco mínimo de ocorrer a perda de confidencialidade das informações, no entanto, os pesquisadores se responsabilizam pela confidencialidade e acesso às mesmas.

Benefícios:
Os potenciais benefícios deste estudo serão a identificação de idosos com alterações de marcha e a realização de uma intervenção com o intuito de aumentar a mobilidade dos idosos e minimizar os riscos de queda.

Comentários e Considerações sobre a Pesquisa:
Esta emenda incorpora as seguintes alterações:

1. PROTOCOLO DE PESQUISA

• ALTERAÇÃO NA EQUIPE DO PROJETO
Razão principal para alteração: Foi incluída uma nova integrante na equipe do projeto, a Enfa. Expedita Angela Henrique, que atua no projeto desde o seu início como assistente de pesquisa e passará a configurar como potencial aluna de pós-graduação.

• AMPLIAÇÃO DO DESFECHO PRIMÁRIO
Razão principal para alteração: Como parte de extensão e ampliação do estudo, o desfecho “medo de queda”, inicialmente selecionado como secundário, passará a ter o status de primário, juntamente com “mobilidade nos espaços de vida”. O objetivo será adequar o método do estudo para que haja poder suficiente para identificar diferenças estatisticamente significativas também quanto a este desfecho.

• AUMENTO DA AMOSTRA
Razão principal para alteração: Com mudança de “medo de queda” para a condição de desfecho

primário, foi realizado novo cálculo amostral, que definiu como 153 sujeitos o tamanho necessário para identificar uma diferença significativa considerando um de 0,05, um poder de 0,80 e uma estimativa de perda de 20% durante o seguimento.

• AUMENTO DO TEMPO DE SEGUIMENTO
Razão principal para alteração: Ainda como parte da extensão e ampliação do estudo, o tempo de seguimento será ampliado de 3 para 6 meses, com entrevistas telefônicas para avaliação dos desfechos de interesse tanto 3 como 6 meses após a inclusão.

As alterações propostas não envolvem riscos adicionais aos pacientes incluídos no estudo, que continua não interferindo nos cuidados clínicos dos sujeitos de pesquisa. Os pacientes incluídos no estudo previamente a aprovação desse adendo serão convidados a assinar novo termo de consentimento referente a entrevista adicional de seguimento de 6 meses.

2. TERMO DE CONSENTIMENTO LIVRE E ESCLARECIDO
Razão principal para alteração: Alterado de acordo com as modificações do protocolo de pesquisa.

3. LISTA DE DOCUMENTOS NESTA EMENDA
- Carta de Justificativa de Emenda, datada de 23 de setembro de 2023;
- Carta de Encaminhamento, datada de 23 de setembro de 2023;
- Projeto de Pesquisa (versão destacado e versão final);
- Termo de Consentimento Livre e Esclarecido (versão destacado e versão final).

Considerações sobre os Termos de apresentação obrigatória:
Realizado as alterações referentes a emenda no Termo de Consentimento Livre e Esclarecido.

Recomendações:
É imprescindível o contato com os participantes que já iniciaram o estudo para a atualização do Termo de Consentimento Livre e Esclarecido. Caso esses participantes não concordem, precisarão ser excluídos do estudo.

Conclusões ou Pendências e Lista de Inadequações:
Emenda do projeto cadastrado no CEPesq como HSL 2023-19, APROVADA nesta data conforme o projeto e o TCLE apresentados.

- Protocolo de Pesquisa
- Termo de Consentimento Livre e Esclarecido

Lembramos que, conforme o item XI.2.d da Res. 466/2012 o pesquisador deverá manter o CEPesq informado sobre o andamento de sua pesquisa através do envio de relatórios parciais (semestrais) e final.

Considerações Finais a critério do CEP:

Este parecer foi elaborado baseado nos documentos abaixo relacionados:

Tipo Documento	Arquivo	Postagem	Autor	Situação
Informações Básicas do Projeto	PB_INFORMAÇÕES_BÁSICAS_2219507_E1.pdf	26/09/2023 23:32:06		Aceito
Folha de Rosto	folhaDeRosto.pdf	26/09/2023 23:31:47	Pedro Kallas Curiati	Aceito
TCLE / Termos de Assentimento / Justificativa de Ausência	TCLEdestacado.docx	25/09/2023 17:01:33	Pedro Kallas Curiati	Aceito
TCLE / Termos de Assentimento / Justificativa de Ausência	TCLE.docx	25/09/2023 17:01:20	Pedro Kallas Curiati	Aceito
Projeto Detalhado / Brochura Investigador	ProjetoEmendadoDestacado.docx	25/09/2023 17:01:07	Pedro Kallas Curiati	Aceito
Projeto Detalhado / Brochura Investigador	ProjetoEmendado.docx	25/09/2023 17:00:52	Pedro Kallas Curiati	Aceito
Solicitação Assinada pelo Pesquisador Responsável	CartaDeEncaminhamentoDaEmenda.pdf	23/09/2023 19:11:19	Pedro Kallas Curiati	Aceito
Outros	CartaDeJustificativaDeEmenda.pdf	23/09/2023 19:10:12	Pedro Kallas Curiati	Aceito
Declaração de Pesquisadores	TermodeCompromisso_ID2999.pdf	10/02/2023 10:45:06	Mirian de Freitas Dal Ben Corradi	Aceito

Situação do Parecer:
Aprovado

Necessita Apreciação da CONEP:
Não

SAO PAULO, 17 de Outubro de 2023

Assinado por:
Mirian de Freitas Dal Ben Corradi
(Coordenador(a))

Endereço: Rua Barata Ribeiro, nº 269
Bairro: Bela Vista **CEP:** 01.308-000
UF: SP **Município:** SAO PAULO
Telefone: (11)3394-5701 **E-mail:** cepesq@hsl.org.br
