# Supplementary material for: Training and provision of mobility aids to promote autonomy and mobility of older patients in a geriatric emergency department: A protocol for a randomized controlled trial
Source: PLoS One. 2024 Jul 31;19(7):e0304397. doi: 10.1371/journal.pone.0304397 (PMC11290684; doi:10.1371/journal.pone.0304397)
Supplement: S4 File — (PDF) [file pone.0304397.s004.pdf]

Ethics Committee Approval Letter

PROJECT DATA

Research title: Training and provision of mobility aids to promote autonomy and mobility of older patients in a geriatric emergency department: a protocol for a randomized controlled trial

Researcher: Pedro Kallas Curiati
Thematic Area:
Version: 3
CAAE (Certificate of Ethical Appreciation): 67316323.9.0000.5461
Proposing Institution: Sociedade Beneficente de Senhoras Hospital Sírio-Libanês
Principal Sponsor: Sociedade Beneficente de Senhoras Hospital Sírio-Libanês

APPROVAL DETAILS

Approval Number: 6.430.440

Project details
The information listed in the fields "Project Presentation", "Research Objective" and "Evaluation of Risks and Benefits" were taken from the Basic Research Information file (PB_INFORMAÇÕES_BÁSICAS_2219507_E1.pdf of 09/26/2023).

Design:
Randomized clinical trial.

ABSTRACT:
INTRODUCTION: Elderly individuals present a higher rate of admission to the emergency department (ED) compared to the younger population. Mobility is the physical ability of a person to move or travel, encompassing both the individual's environment and their ability to adapt to it. Reduced mobility leads to decreased social interaction, isolation, and loneliness, affecting the physical, psychological, and social aspects of the elderly. Mobility aids can be used to improve mobility and prevent falls, and international guidelines recommend their availability in the ED environment. OBJECTIVES: Evaluate the effectiveness of a training program and provision of mobility aids, with or without telemonitoring, on functional mobility, balance, gait speed, quality of life, and fear of

falls and Fall Count in Elderly Patients Treated in the Emergency Department: METHODS: A randomized clinical trial will be conducted, recruiting elderly individuals receiving care at the Emergency Department (ED) of Hospital Sírio Libanês (HSL). Inclusion criteria are: age 65 years or older; ED attendance at HSL; at least one indication for the use of walking aids; and signing of the Informed Consent Form (ICF). Exclusion criteria include: altered level of consciousness; need for supplemental oxygen (3L/min); respiratory discomfort; hemodynamic instability; postural instability; cognitive impairment hindering the use of walking aids; hospitalization after initial ED assessment; delirium. Participants will be randomized into three intervention groups: A) Walking Aid Group (WAG), where patients will receive training with walking aids and guidance on safe walking, but no telemonitoring; B) Walking Aid and Telemonitoring Group (WAG+Tele), where patients will receive training with walking aids, guidance on safe walking, and telemonitoring; C) Control Group (CG), where patients will only receive guidance on safe walking and neither training with walking aids nor telemonitoring. Participants will be assessed for age, gender, social and demographic data, clinical data, mobility in living spaces, gait speed, muscle strength, functionality, quality of life, fear of falling, fall history, and cognitive and psychosocial evaluation. Baseline assessment will occur before intervention. Gait speed and fear of falling assessments will be repeated post-intervention. Additionally, functionality, quality of life, fear of falling, fall history, cognition, and psychosocial status will be reassessed 3 months after discharge from geriatric emergency care through a telephone interview. Primary outcome will be mobility in living spaces, with secondary outcomes including gait speed, functionality, muscle strength, quality of life, fear of falling, and occurrence of falls within 3 months. Statistical analysis will employ paired t-test, Wilcoxon test, One-Way ANOVA, Kruskall-Wallis, Pearson correlation, and Spearman test according to normality established by Shapiro-Wilk normality test. Significance will be considered at $P<0.05$. Introduction: According to data from the United Nations (UN) and the World Health Organization (WHO), Brazil and the world have experienced a pronounced increase in their elderly population

during the last two decades (1), the United Nations (UN), through its Department of Economics and Social Affairs (DESA), conducted a study to better understand the impact of population aging on society, the environment, and the global economy. The study identified Brazil and the South American continent as prominent examples of this demographic transition (2). Using graphs, DESA illustrated the increase in the fraction of elderly individuals in the Brazilian population, which was less than 5% in 1990, currently ranging between 7% and 9%, and estimated to surpass 15% by 2050 (2).

In this context, the emergency department (ED) plays a significant role for the elderly population, serving as a center for emergency treatment, an entry point for acute and highly complex care, access to continuous healthcare services, a readily available 24-hour medical care source, and a safety net when the smooth transition of care between healthcare systems is compromised (3, 4). According to the National Center for Health Statistics, a department of the Centers for Disease Control and Prevention (CDC) in the United States, individuals aged over 74 represented 56.7% of the annual demand for ED services in 2018 (5). In Switzerland, between 2002 and 2012, emergency visits by individuals aged 60 or older increased by 42.3%, compared to only 27.6% in younger age groups (6).

Due to their multiple and interconnected medical and social problems, older adults often challenge systems of care typically developed for individuals with single-system disorders and robust social support networks (7) (8). When compared to younger populations, geriatric patients are more frequently admitted to EDs brought in by ambulances, triaged as emergencies, and directed for hospitalization (9) (10). In 2015, in the United States, 29% of visits by individuals aged 75 or older resulted in hospitalization with an average stay of 5 days, while only 5% of visits by adults aged 25 to 44 had the same outcome (9). In 2018, 16.1% of elderly ED visits in the United States were classified as emergencies, 40.2% as urgent, and 17.7% culminated in hospitalization (11).

Despite older adults undergoing more tests and procedures than younger patients, their diagnoses tend to be less precise, attributed to a higher frequency of atypical disease presentations, polypharmacy, and multimorbidities (3, 7-10, 12-19). As a result, emergency physicians report greater difficulty in managing elderly patients (3, 20), and many older adults complain about the lack of resolution of their complaints in ED services (3, 21, 22). Additionally, visits to

The provision of PA (Primary Care) services in this population is associated with high rates of admission to acute care units, prolonged hospitalization, unplanned hospital readmission, functional dependence, and mortality (7, 10, 15, 23-26). However, it would be overly simplistic to attribute the worst prognosis for the elderly strictly to clinical factors, as it is necessary to consider that healthcare systems may be less prepared to meet their needs (10, 22). Strategies for differentiated care for the elderly may include programs with comprehensive geriatric assessment models, care transition teams, facilitated discharge protocols, day hospital care, home care, and admissions to geriatric acute care units (10, 27).

Comprehensive Geriatric Assessment (CGA) is an interdisciplinary and multidimensional diagnostic process aimed at determining the clinical, functional, and psychological status of frail older adults in order to develop a long-term treatment and monitoring plan (27, 28). The benefits of implementing CGA include improved cognitive performance, reduced functional loss, lower institutionalization and mortality rates, and a decrease in unnecessary hospitalizations and length of hospital stay (10, 29-33). According to international guidelines, the care of the elderly in the PA requires differentiated policies, protocols, and workflows designed for the specific characteristics of this population (12).

The European guideline highlights the "5 Ms of geriatrics" as the main recommendations for this population: mind, medications, multicomplexity, "most important," and mobility. In other words, when caring for the elderly in the PA service, it is recommended to address dementia, delirium, depression, and cognitive impairment; conduct a structured assessment and review of medications used by the elderly, considering potential drug interactions and inappropriate use of medications; weigh the general needs of the elderly, whether medical, psychological, social, functional, or environmental; and regarding the "most important" aspect, ensure individualized health outcomes focusing on goals that are meaningful to the elderly (34).

It is also recommended that the physical space of the geriatric PA focus on structural modifications aimed at safety, comfort, memory cues, and sensory perception (both vision and hearing), ensuring greater mobility for the elderly. Additionally, the guidelines emphasize that improvements in mobility and safety are related not only to furniture but also underscore the importance of easy access to assistive walking devices (12).

Mobility is a term used not only to describe a person's physical ability to move or travel but also encompasses the person's environment and their ability to adapt to it (35). It is directly related to the individual's functionality, independence, autonomy, and quality of life (35) and can be evaluated both by

a measure of an individual's ability to transfer from furniture (bed or chair), walk, and climb stairs, as well as their living space, defined by the distance they can travel within and outside the home, with or without assistance (35). Mobility limitations are common in the elderly and are associated with depressive symptoms and decreased quality of life, leading to reduced social interaction, isolation, and loneliness, affecting the physical, psychological, and social aspects of the elderly (36). Risk factors most associated with mobility limitation include advanced age, low physical activity, obesity, changes in strength and balance, gait disturbances, chronic diseases, and other less reported factors such as depressive symptoms, cognitive deficits, alcohol consumption, smoking, and recent hospitalization (35). Therefore, the assessment of mobility should be considered a component to be included in elderly healthcare (35).

Correlated with mobility, the term "life space" is currently used, an emerging concept related to functional, environmental, and social factors that define how people experience their daily lives (37). A systematic review published in 2019 revealed that life space mobility shows a moderate association with cognitive function, predicting the execution of actions, learning, memory, attention, visuospatial ability, and perceptuomotor function. These cognitive function factors are linked to the autonomy of the elderly, and both mobility and cognitive function may decline during aging (38). In this context, the University of Alabama at Birmingham developed a low-cost instrument in 2003 that does not require specific space, equipment, or training and can be applied either in person or via telephone contact: the Life Space Assessment (LSA) (39). This instrument was validated in a Brazilian study in 2018, evaluating 80 individuals aged 60 years and older recruited from public health services (outpatient and home care) (39). It was concluded that the LSA has adequate reliability, validity, and reproducibility for assessing mobility in the life spaces of Brazilian elderly individuals, with Cronbach's alpha = 0.92 and ICC = 0.97, similar to the original version (ICC = 0.96) (39).

The LSA evaluates the elderly's mobility in life spaces in five different scenarios in the four preceding weeks, with a description of frequency and independence for each level (39). Subscores for life space mobility are obtained for each life space level (1-5) by multiplying frequency and independence. Subscores from each level are then added to obtain the final LSA score, ranging from 0 to 120, where 0 indicates an individual restricted to the room where they sleep, and 120 indicates an individual who independently travels daily to other cities (40, 41). A study demonstrated that the cutoff score for the risk of decline in instrumental activities of daily living would be 56 points (42). The LSA is

A tool that recognizes that cognitive and functional factors affect mobility, gait quality (rhythm, speed, gait variability), and an individual's ability to perform daily activities (43). A study conducted in 2016 in the United States followed individuals aged 75 and above for 3 years through an initial home interview and monthly telephone interviews with the aim of assessing the association between visits to the PA service and the need for hospitalization with community mobility (44). Mobility was assessed using the LSA tool, and reasons for admissions, patient comorbidities, and daily activities were recorded using the Katz index (44). It was observed that elderly individuals who required a visit to the PA service (about 20% of participants) or hospitalization (about 40%) had a lower LSA score, which did not return to the pre-contact value even after a year of health service contact (44).

Mobility aids promote independence within the environment through biomechanical stabilization, improved balance and motor control, sensory feedback, reduced lower limb load, increased confidence, and fall prevention (45, 46). However, they may increase the risk of falls, both due to improper use and prescription for patients with balance deficits and previous gait alterations (45). For this reason, it is recommended that their use be prescribed by a qualified professional and be preceded by guidance, training, and monitoring for the optimization of benefits, risk reduction, and increased adherence and confidence (45, 46).

Several studies point to telemonitoring as a tool for healthcare (47). It is defined as the use of information technology and telecommunications for distance healthcare (48). In a systematic review published in 2019, it was considered feasible and well-received for elderly healthcare and recommended for clinical practice for overcoming distance and access barriers to healthcare services (49). Additionally, a study conducted in 2021 with 60 frail elderly individuals compared the care of this population over a year, with one group using telemonitoring with a multiprofessional team and the other receiving conventional care, demonstrating better results for telemonitoring in terms of mood, behavior, daily activities, and nutritional status (50).

Current literature on telemonitoring in the elderly population and potential physiotherapy interventions includes telemonitoring and telerehabilitation for elderly individuals with chronic obstructive pulmonary disease (COPD) and heart diseases, with evidence of effectiveness for home treatment continuation in rehabilitation programs after hospitalizations (acute events and postoperative), promotion of rehabilitation, improvement of functional capacity, and reduction of readmissions (51-53). Few studies have explored telemonitoring for the PA service population, despite evidence that

Intervention in elderly individuals with multiple diseases reduces the number of hospitalizations and visits to the Emergency Department (54, 55). In fact, there are no studies evaluating the effects of an intervention using walking aids, with or without telemonitoring, on the mobility of elderly individuals admitted to the Emergency Department. Acutely ill elderly individuals admitted through the Emergency Department with complaints associated or not with falls have a higher risk of falls, impacting functionality, quality of life, cognition, revisits, and mortality (28). Therefore, it is essential to incorporate mobility screenings and assessments, gait quality, and fall risk in elderly individuals within Emergency Departments and implement measures to promote safety, mobility, and quality of life for these patients, including the appropriate and safe use of walking aids. It is worth noting that, to date, there is no study proving their safety or benefit in acute care settings, despite recommendations from international guidelines and Geriatric Emergency Department Accreditation (GEDA) (12).

Hypothesis:
A training program and provision of walking aids, with or without telemonitoring, promote improvement in mobility and functional balance in elderly individuals attending Emergency Department services. A training program and provision of walking aids, with or without telemonitoring, promote improvement in walking speed, quality of life, fear of falling, and the number of falls within 3 months in elderly individuals attending Emergency Department services.

Proposed Methodology:
- Population:
Elderly individuals receiving care in the Emergency Department of HSL will be recruited.

Interventions with an equal number of patients, which may include:
1) Walking Aid Group (WAG)
2) Walking Aid Group with Telemonitoring (WAG+Tele)
3) Control Group (CG)

- Groups:
 - Walking Aid Group (WAG): Patients will receive training with the walking aid and guidance on safe walking but will not receive telemonitoring.

• Gait Assist Device and Telemonitoring Group (GDAM+Tele): Patients will receive training with the gait assist device, guidance on safe walking, and telemonitoring.
• Control Group (GC): Patients will only receive guidance on safe walking and will not undergo training with the gait assist device or telemonitoring.

- Blinding

The study will be blinded for the statistician. The data will be analyzed by a researcher who did not participate in the interventions or evaluations. Additionally, the researcher conducting the final project evaluation will also be blinded. The groups will be coded, and it will not be possible to identify membership in GDAM, GDAM+Tele, or GC.

Procedures

- Recruitment

Participants will be recruited at the HSL PA, Bela Vista Unit, São Paulo, Brazil, by a trained research assistant who will be on duty for 25 hours per week in shifts between 7:00 and 18:00 for up to 6 months. The assistant may be called upon by the medical and/or nursing team, but they will also actively search for potential study candidates. Eligible patients for the study screening will be contacted by the research team for the screening process and signing of the Informed Consent Form (ICF).

- Screening

Initially, all voluntary participants will undergo a screening process to ensure compliance with eligibility criteria in this study. At this stage, participants will undergo an initial interview to collect sociodemographic, clinical, and medication data. Another factor to be assessed and considered as an exclusion criterion is delirium, defined as an organic brain syndrome characterized by disturbance in consciousness, attention, perception, thinking, memory, psychomotor behavior, emotion, and sleep-wake cycle (58, 59). The Confusion Assessment Method (CAM) will be used for this evaluation, a rapid tool that can be applied by clinicians and researchers without the need for psychiatric training (60). This tool has been validated for the elderly population in PA services, initially in Canada and later in a Brazilian study (61-63). There are two versions for its use, the short version with 4 items used for screening, and the long version widely used for diagnostic confirmation, classification, and research purposes (60).

- Evaluative Timepoints

All baseline assessments will be conducted before the intervention. The gait speed and fear of falling assessment will be repeated after the intervention. The assessment of mobility in living spaces, functionality, quality of life, fear of falling, number of falls, and cognition will be repeated 3 months after the intervention through a telephone interview or video call.

Inclusion Criteria:

The following inclusion criteria will be adopted:

- Age equal to or greater than 65 years;
- Attendance at HSL's PA;
- Meeting at least one criterion from the institutional protocol for the indication and training of gait assist devices in the PA (Appendix 1): increased postural stability; increased somatosensory feedback; aid in motor control; reduction of biomechanical overload; promotion of autonomy with safety; and a history of falls (last six months).
- Signing the Informed Consent Form (ICF).

Exclusion Criteria:

The following criteria will be considered for study exclusion:

- Altered level of consciousness;
- Need for supplemental oxygen (3L/min);
- Respiratory discomfort; Hemodynamic instability;
- Postural instability with a tendency to fall backward;
- Cognitive impairment that hinders the manipulation of the gait assist device;
- Hospitalization after evaluation in the PA service;
- Delirium.

Data Analysis Methodology:

Continuous variables will be expressed as mean and standard deviation (SD) or median and interquartile range (IQR) 25%-75%. Categorical data will be presented in absolute number and relative (%) terms. To check the normality distribution of the data, the Shapiro-Wilk normality test will be applied. Paired t-tests will be used for parametric data, and the Wilcoxon test for non-parametric data. For the evaluation between the three groups,

In the intervention, One-Way ANOVA will be used for parametric data, or Kruskall-Wallis for non-parametric data. To assess correlations, Pearson correlation tests will be employed for parametric data, and Spearman's test for non-parametric data. All analyses will be conducted using the Statistical Package for Social Sciences (SPSS) software, version 28.0.1 (SPSS Inc.®, Chicago, IL, USA), with a significance level of 5%.

Primary Outcome:
- Mobility in living spaces.

Secondary Outcomes:
- Gait speed;
- Functionality;
- Quality of life;
- Concerns about falling;
- Incidence of falls in 3 months.

Objective
Primary Objective:
To assess the effectiveness of a training program and provision of walking aids, with or without telemonitoring, on the mobility and functional balance of elderly individuals receiving care at a primary care service.

Secondary Objective:
To evaluate the effectiveness of a training program and provision of walking aids, with or without telemonitoring, on gait speed, quality of life, fear of falling, and the number of falls within a 3-month period among elderly individuals receiving care at a primary care service.

Risks:
Voluntary participants in this study may report varying degrees of discomfort, fatigue, or tiredness, especially during the execution of functional and strength tests.

Application of the interview. However, these symptoms should subside after 5 minutes of rest. All collected information will be compiled and managed through the electronic data capture system (REDCap) hosted on the servers of Hospital Sírio-Libanês. The data will be made available to researchers in a "de-identified" manner to protect the participant's identity and ensure data integrity. Database access will be limited to the investigators comprising the research team. There is a minimal risk of confidentiality loss; however, researchers take responsibility for maintaining confidentiality and controlling access.

Benefits:
The potential benefits of this study include identifying elderly individuals with gait alterations and implementing an intervention to enhance their mobility while minimizing the risk of falls.

Comments and Considerations on the Research:

"This amendment incorporates the following changes:

1. RESEARCH PROTOCOL

- CHANGES IN PROJECT TEAM

Main reason for change: A new member, Nurse Expedita Angela Henrique, has been added to the project team. She has been involved in the project since its inception as a research assistant and will now be considered a potential graduate student.

- EXPANSION OF PRIMARY OUTCOME

Main reason for change: As part of the study's extension and expansion, the outcome 'fear of falling,' initially selected as secondary, will now be designated as primary, along with 'mobility in living spaces.' The aim is to adjust the study's methodology to have sufficient power to identify statistically significant differences in this outcome as well.

- INCREASE IN SAMPLE SIZE

Main reason for change: With the shift of 'fear of falling' to a primary outcome

Primary, a new sample calculation was performed, which defined 153 subjects as the necessary size to identify a significant difference considering an alpha of 0.05, a power of 0.80, and an estimated 20% loss during follow-up.

INCREASE IN FOLLOW-UP TIME

Main reason for the change: Still as part of the extension and expansion of the study, the follow-up time will be extended from 3 to 6 months, with telephone interviews to assess outcomes of interest at both 3 and 6 months after inclusion.

The proposed changes do not involve additional risks to the patients included in the study, which continues not to interfere with the clinical care of the research subjects. Patients included in the study prior to the approval of this amendment will be invited to sign a new consent form regarding the additional 6-month follow-up interview.

2. INFORMED CONSENT FORM

Main reason for the change: Altered according to the modifications of the research protocol.

3. LIST OF DOCUMENTS IN THIS AMENDMENT

- Letter of Justification for Amendment, dated September 23, 2023;
- Cover Letter, dated September 23, 2023;
- Research Project (highlighted version and final version);
- Informed Consent Form (highlighted version and final version).

Considerations about Mandatory Submission Terms:

Changes regarding the amendment have been made in the Informed Consent Form. Considerations about mandatory presentation terms:

Recommendations:

It is essential to contact participants who have already started the study to update the Terms of Reference Free and Informed Consent. If these participants do not agree, they will need to be excluded from the
study.

Conclusions or pending issues and list of inadequacies:

Amendment of the project registered with CEPesq as HSL 2023-19, APPROVED on this date according to the project and the TCLE presented.

- Research Protocol
- Free and Informed Consent Form

We remind you that, according to item XI.2.d of Res. 466/2012, the researcher must maintain the CEPesq informed about the progress of your research by sending partial (half-yearly) and final reports.

Final Considerations at the discretion of the CEP:

This opinion was prepared based on the documents listed below: - Research Protocol

- Free and Informed Consent Form

We remind you that, according to item XI.2.d of Res. 466/2012, the researcher must maintain the CEPesq informed about the progress of your research by sending partial (half-yearly) and final reports.

Final Considerations at the discretion of the CEP:

This opinion was prepared based on the documents listed below:

Document Type	File Name	Data	Author	Opinion
Basic information from the project	PB_INFORMAÇÕES_BÁSICAS_2219507_E1.pdf	26/09/2023 23:32:06		accepted
Title Page	folhaDeRosto.pdf	26/09/2023 23:31:47	Pedro Kallas Curiati	accepted
TCLE / Terms of Assent / Justification of Absence	TCLEdestacado.docx	25/09/2023 17:01:33	Pedro Kallas Curiati	accepted
TCLE / Terms of Assent / Justification of Absence	TCLE.docx	25/09/2023 17:01:20	Pedro Kallas Curiati	accepted
Detailed project / Brochure Investigator	ProjetoEmendadoDestacado.docx	25/09/2023 17:01:07	Pedro Kallas Curiati	accepted
Detailed project / Brochure Investigator	ProjetoEmendado.docx	25/09/2023 17:00:52	Pedro Kallas Curiati	accepted
Signed Request by Researcher Responsible	CartaDeEncaminhamentoDaEmenda.pdf	23/09/2023 19:11:19	Pedro Kallas Curiati	accepted
Others	CartaDeJustificativaDeEmenda.pdf	23/09/2023 19:10:12	Pedro Kallas Curiati	accepted
Declaration of Researchers	TermodeCompromisso_ID2999.pdf	10/02/2023 10:45:06	Mirian de Freitas Dal Ben Corradi	accepted

Status:

Approved

Requires CONEP Appraisal:

No

SAO PAULO, October 17, 2023

Assinado por:
Mirian de Freitas Dal Ben Corradi
(Coordenador(a))
