# Supplementary material for: Training and provision of mobility aids to promote autonomy and mobility of older patients in a geriatric emergency department: A protocol for a randomized controlled trial
Source: PLoS One. 2024 Jul 31;19(7):e0304397. doi: 10.1371/journal.pone.0304397 (PMC11290684; doi:10.1371/journal.pone.0304397)
Supplement: S5 File — (PDF) [file pone.0304397.s005.pdf]

PORTUGUESE VERSION

Treinamento e fornecimento de dispositivos auxiliares de marcha para redução do medo de queda e promoção de autonomia e mobilidade de idosos em pronto atendimento: um ensaio clínico randomizado.

Pesquisadores responsáveis:

Fernanda Sato Polesel

Sâmia Denadai

Marlon Juliano Romero Aliberti

Christian Valle Morinaga

Mario Chueire de Andrade-Junior

Itiana Cardoso Madalena

Wellington Pereira Yamaguti

Pedro Kallas Curiati

Renato Fraga Righetti

Hospital Sírio-Libanês, São Paulo – SP, Brasil.

RESUMO

INTRODUÇÃO: Dispositivos auxiliares de marcha podem ser utilizados para reduzir o medo de cair e aumentar a mobilidade e prevenir quedas em idosos. Segundo diretrizes internacionais, devem estar disponíveis no ambiente de pronto atendimento (PA) geriátrico. **OBJETIVOS:** Avaliar a eficácia de um programa de treinamento e fornecimento de dispositivos auxiliares de marcha, associado ou não a telemonitoramento, no medo de queda e seu impacto na mobilidade e equilíbrio funcional, assim como velocidade de marcha, qualidade de vida e número de queda em 3 e 6 meses, em idosos atendidos em serviço de PA. **MÉTODOS:** Será realizado um ensaio clínico randomizado em que serão recrutados indivíduos idosos em atendimento no PA do Hospital Sírio Libanês (HSL). Os critérios de inclusão serão: idade superior ou igual a 65 anos; atendimento no PA do HSL; ao menos 1 indicação para uso de dispositivos auxiliares da marcha. Os critérios de exclusão incluem: alteração do nível de consciência; necessidade de oxigênio suplementar (≥3L/min); desconforto respiratório; instabilidade hemodinâmica; instabilidade postural; comprometimento cognitivo que dificulte o uso de dispositivo auxiliar de marcha; internação após avaliação inicial no serviço de PA; delirium. Os participantes serão randomizados e alocados em três grupos de intervenção, sendo: A) Grupo dispositivo auxiliar de marcha (GDAM), em que os pacientes receberão treinamento com dispositivo auxiliar de marcha e orientações sobre marcha segura, mas não receberão telemonitoramento; B) Grupo dispositivo auxiliar de marcha e telemonitoramento (GDAM+Tele), em que os pacientes receberão treinamento com o dispositivo auxiliar de marcha, orientações sobre marcha segura e telemonitoramento; C) Grupo controle (GC), em que os pacientes receberão somente orientações sobre marcha segura e não receberão nem treinamento com o dispositivo auxiliar de marcha nem telemonitoramento. Será considerado como desfecho primário o medo de cair e a mobilidade nos espaços de vida e como desfechos secundários velocidade de marcha, funcionalidade, força muscular, qualidade de vida, preocupação com a queda e ocorrência de queda em 3 e 6 meses. Para análise estatística, serão utilizados teste t pareado, teste de Wilcoxon, One-Way ANOVA, Kruskall-Wallis, correlação de Pearson e teste de Spearman de acordo com a normalidade estabelecida pelo teste de normalidade Shapiro Wilk. Será considerado significante quando $P<0,05$.

Palavras-chave: idosos, mobilidade, dispositivo auxiliar de marcha, pronto atendimento

INTRODUÇÃO

De acordo com as diretrizes internacionais, o atendimento do idoso no pronto atendimentos demanda políticas, protocolos e fluxos diferenciados, projetados para as particularidades dessa população (1). A diretriz europeia cita como principais recomendações para este público os “5 Ms da geriatria”: mente, medicamentos, multicomplexidade, “mais importante” e mobilidade. Em outras palavras, durante o atendimento do idoso no serviço PA, recomenda-se: abordagem de demência, delirium, depressão e comprometimento cognitivo; avaliação estruturada e revisão dos medicamentos que são utilizados pelos idosos, considerando possíveis interações medicamentosas e uso inapropriado de medicamentos; ponderação das necessidades gerais do idoso, sejam elas médicas, psicológicas, sociais, funcionais ou ambientais; quanto ao item “mais importante” recomenda-se garantir os resultados de saúde de maneira individualizada focando nos objetivos que tenham significado para o idoso (2). Recomenda-se também que o espaço físico do PA geriátrico foque em modificações estruturais que visem a segurança, conforto, pistas de memória e percepção sensorial (tanto visão quanto audição), garantindo maior mobilidade dos idosos. Além disso, as diretrizes destacam que as melhorias para mobilidade e segurança, estão relacionados não apenas ao mobiliário, mas também reforçam a importância do fácil acesso a dispositivos auxiliares de marcha (1).

As limitações de mobilidade são comuns nos idosos e estão associadas a sintomas depressivos e diminuição da qualidade de vida, visto culminam em diminuição de convívio social, isolamento e solidão, afetando aspectos físicos, psicológicos e sociais do idoso (3). Os fatores de risco que estão mais associados à limitação de mobilidade são idade avançada, baixa atividade física, obesidade, alteração de força e equilíbrio, alterações de marcha e doenças crônicas, além de outros fatores menos relatados como sintomas depressivos e déficit cognitivo, ingestão de álcool, tabagismo e hospitalização recente (4). Portanto, a avalição de mobilidade deve ser considerada um componente a ser incluído no cuidado de saúde do idoso (4).

As intervenções com dispositivos auxiliares de marcha promovem a independência dentro do ambiente através de estabilização biomecânica, melhora do equilíbrio e do controle motor, feedback sensorial, redução da carga dos membros inferiores, redução do medo de queda e prevenção de quedas (5, 6). Além disso, diversos estudos apontam o telemonitoramento como uma ferramenta para o cuidado a saúde (7). Ele é definido como o uso de tecnologia da informação e telecomunicações para o cuidado a saúde à distância (8). Em uma revisão sistemática publicada em 2019, foi considerado viável e bem recebido para o cuidado da saúde do idoso e recomendado para a prática clínica por superar barreiras de distância e acesso a serviços de saúde (9). Poucos estudos exploraram o

telemonitoramento para a população no serviço de PA, apesar da evidência de que essa intervenção em idosos com múltiplas doenças reduz o número de internações e visitas ao PA (10, 11). De fato, não há estudos que avaliem os efeitos de uma intervenção de dispositivos de auxílio a marcha, associado ou não ao telemonitoramento, na mobilidade de idosos admitidos ao serviço de PA.

Baseado no projeto de treinamento e fornecimento de dispositivos auxiliares de marcha para promoção de mobilidade de idosos no pronto atendimento previamente aprovado e em andamento, esse novo projeto proposto trata-se de uma extensão em que desfecho primário de medo de cair e avaliação em 3 e 6 meses foram adicionados.

OBJETIVOS

Objetivo Primário

Avaliar a eficácia de um programa de treinamento e fornecimento de dispositivos auxiliares de marcha, associado ou não a telemonitoramento, no medo de cair, mobilidade e no equilíbrio funcional de idosos atendidos em serviço de PA.

Objetivo Secundário

Avaliar a eficácia de um programa de treinamento e fornecimento de dispositivos auxiliares de marcha, associado ou não a telemonitoramento, qualidade de vida, número de queda em 3 e 6 meses em idosos atendidos em serviço de PA.

MÉTODOS

Delineamento

Inicialmente, a emenda do projeto de pesquisa será submetida ao Comitê de Ética em Pesquisa (CEP) do Hospital Sírio-Libanês. O protocolo deste projeto de pesquisa será registrado na plataforma do "*ClinicalTrials.gov*" como forma de prover informações a respeito deste ensaio clínico, bem como garantir transparência na condução das etapas deste estudo.

Desenho do estudo

Estudo experimental do tipo ensaio clínico randomizado e cego. Será conduzido nas dependências do Hospital Sírio-Libanês (HSL) – Unidade Bela Vista, instituição localizada no município de São Paulo, SP – Brasil.

População

Serão recrutados indivíduos idosos que estarão em atendimento no PA do HSL.

Cálculo amostral

O cálculo da amostra foi baseado no estudo de Morgan e cols. de 2014 (12), que descreveu uma diferença de 8,2 no medo de cair (FES-I), com um desvio padrão de 12 pontos. Para um α de 0,05 e um poder de 0,80, o tamanho total da amostra foi definido em 42 indivíduos. Diante de uma estimativa de perda de 20% durante o seguimento, a amostra necessária para o sucesso do estudo foi ampliada para 51 indivíduos por grupo, totalizando 153 participantes.

Critérios de elegibilidade

Para os pacientes que assinarem o Termo de Consentimento Livre e Esclarecido (TCLE) serão adotados os seguintes critérios de inclusão: idade superior ou igual a 65 anos; atendimento no PA do HSL; preenchimento de pelo menos um critério do protocolo institucional para indicação e treinamento de dispositivos auxiliares da marcha no PA: aumento da estabilidade postural; aumento do retorno somatossensorial; auxílio no controle motor; redução da sobrecarga biomecânica; promoção da autonomia com segurança; e histórico de quedas (seis últimos meses).

Critérios de exclusão

Serão considerados para exclusão do estudo os seguintes critérios: alteração do nível de consciência; necessidade de oxigênio suplementar (≥3L/min); desconforto respiratório;

instabilidade hemodinâmica; instabilidade postural com tendência de queda para trás; comprometimento cognitivo que prejudique manipulação do dispositivo auxiliar de marcha; internação após avaliação no serviço de PA; delirium.

Randomização e alocação

O processo de randomização será realizado por meio do REDCap, garantindo uma chance semelhante de ser alocado em qualquer um dos grupos de intervenção (1:1:1). A randomização irá determinar a alocação em três grupos, podendo ser: 1) Grupo dispositivo auxiliar de marcha (AM); 2) Grupo dispositivo auxiliar de marcha com telemonitoramento (GDAM+Tele); 3) Grupo Controle (GC).

Grupos

- **Grupo dispositivo auxiliar de marcha (GDAM):** os pacientes receberão treinamento com o dispositivo de marcha e orientações sobre marcha segura, mas não receberão telemonitoramento;
- **Grupo dispositivo auxiliar de marcha e telemonitoramento (GDAM+Tele):** os pacientes receberão treinamento com o dispositivo de marcha, orientações sobre marcha segura e telemonitoramento;
- **Grupo controle (GC):** os pacientes receberão somente orientações sobre marcha segura e não receberão treinamento com o dispositivo de marcha nem telemonitoramento.

Cegamento

O estudo será cego para o avaliador que realizará as avaliações de 3 e 6 meses e o estatístico.

Procedimentos

Recrutamento

Os participantes serão recrutados no PA do HSL, Unidade Bela Vista, São Paulo, Brasil, por assistente de pesquisa treinado, que permanecerá de plantão por 25 horas semanais em turnos distribuídos entre 7:00 e 18:00 por até 6 meses. Este poderá ser

acionado pela equipe médica e/ou de enfermagem, mas também fará busca ativa de potenciais candidatos para o estudo. Os pacientes elegíveis para a triagem do estudo serão contatados pela equipe de pesquisa para sua realização e para assinatura do Termo de Consentimento Livre e Esclarecido (TCLE).

Triagem

Inicialmente, todos os participantes voluntários serão submetidos a um processo de triagem para assegurar o cumprimento dos critérios de elegibilidade neste estudo. Nesta etapa, os participantes serão submetidos a uma entrevista inicial para coleta de dados sociodemográficos, clínicos e de medicamentos. Além disso, o delirium será considerado critério de exclusão (13, 14). Para esta avaliação será utilizado a *Escala Confusion Assessment Method* (CAM) (15).

Momentos avaliativos

Toda a avaliação basal será realizada antes da intervenção. A avaliação de velocidade da marcha e medo de cair será repetida após a intervenção. A avaliação de mobilidade nos espaços de vida, funcionalidade, qualidade de vida, medo de cair, número de quedas e cognição será repetida 3 e 6 meses após a intervenção por entrevista telefônica ou videochamada.

Medo de cair

A preocupação relacionada à queda será avaliada pela *Falls Efficacy Scale International* (FES-I) (16). O questionário é composto por 16 itens, que avaliam, por exemplo, caminhar em superfícies escorregadias, irregulares ou inclinadas, visitar amigos ou parentes e ir a um evento social (17). A preocupação com a queda na realização de cada atividade é classificada em uma escala de quatro pontos (intervalo 1 = nada preocupado a 4 = muito preocupado) (17). A pontuação total da FES-I varia de 16 a 64, com valores de corte: 16-22 (preocupação baixa), 20-27 (preocupação moderada) e 28-64 (preocupação alta) (17).

Mobilidade nos espaços de vida

Será realizada pela LSA, que permite caracterizar a mobilidade nos espaços de vida (outros cômodos da residência que não o dormitório, ambiente externo da residência, redondezas/ bairro, fora do bairro, fora da cidade) quanto a frequência, necessidade de

dispositivo de auxílio a marcha e necessidade de ajuda de terceiros durante as últimas 4 semanas (18). A pontuação composta reflete a distância, a frequência e o nível de independência percorrido, com intervalo de 0 a 120 (19).

Avaliação do Tempo da marcha

O teste *Timed Up and Go* (TUG) permite avaliar mobilidade, equilíbrio, habilidade de marcha e risco de queda (20). Para a realização do teste, será solicitado que o indivíduo levante da cadeira sem a utilização dos braços, percorra um trajeto de 3 metros, dê a volta por fora de um cone (equipe de fisioterapia), retorne em direção a cadeira e sente sem a utilização dos braços (21).

Força muscular periférica

A força muscular periférica será mensurada por meio da medida da força de preensão manual (FPM) utilizando um dinamômetro hidráulico manual (modelo SH 5001, marca SAEHAN) (22), respeitando-se o protocolo recomendado pela *American Association of Hand Therapists* (ASHT) (23). A melhor marca dentre três avaliações aceitáveis será considerada como a medida da força de preensão manual. Para tal avaliação será utilizado o dinamômetro da equipe de pesquisa da fisioterapia do HSL.

Funcionalidade

O teste de sentar e levantar de 1 minuto será realizado com base no protocolo previamente descrito por Ozalevli e cols. (24). Os sujeitos serão orientados a levantar e sentar completamente em uma cadeira quantas vezes for possível (altura = 46 cm) durante 1 min (24). Para avaliação das atividades básicas de vida diária (ABVD), será utilizado o Índice de Katz (25). Como resultado, o indivíduo pode ser classificado de 0 pontos (independência para ABVD) a 6 pontos (dependência total para ABVD), passando por 1 a 5 pontos (dependência parcial para ABVD) (26, 27). O Índice de Barthel, além de avaliar a autonomia no cuidado pessoal, avalia também a mobilidade (28-30). A pontuação final varia de 0 a 100, sendo que cada item é pontuado de acordo com a maneira como o indivíduo executa cada tarefa, seja ela de maneira independente, com alguma ajuda ou de maneira dependente (28-30). As pontuações finais mais elevadas indicam uma maior independência: 80 a 100 indica independência; 60 a 79 pontos, dependência leve; 40 a 59 pontos, dependência moderada; 20 a 39 pontos, dependência grave; menos de 20 pontos, dependência total (28-30). Atividades instrumentais de vida diária (AIVD) serão avaliadas com uma escala criada em

1969 por Lawton e Brody, em que sete atividades são incluídas: usar o telefone, locomover-se através de meio de transporte, fazer compras, realizar trabalhos domésticos, preparar refeições, utilizar medicamentos e manejar finanças (31). A pontuação final permite classificar os indivíduos em independentes (25 a 27 pontos), dependentes leves (21 a 25 pontos), dependentes moderados (16 a 20 pontos), dependente graves (10 a 15 pontos) e totalmente dependentes (9 pontos) (31).

Qualidade de vida

Para a avaliação da qualidade de vida será utilizado o Euro Quality of Life Instrument – 5D (EQ-5D) (32). O EQ-5D contempla cinco domínios de saúde: mobilidade; autocuidado; atividades cotidianas; dor/desconforto; e ansiedade/depressão (33). Cada domínio é classificado em três níveis: sem problemas (1 ponto); alguns problemas (2 pontos); e problemas extremos (3 pontos) (33). Por fim, prevê uma autoavaliação da saúde em escala análogo-visual (EAV), que vai de 0 (pior saúde possível) a 100 (melhor saúde possível), resultando em 243/3125 estados de saúde distintos (32).

Histórico de quedas

O histórico de quedas será avaliado periodicamente durante o estudo e os pacientes serão convidados a preencher um diário detalhado dos eventos em todos os momentos em que houver uma queda (34). Para cada queda, serão discriminados localização, lesões associadas e necessidade de cuidados especiais devido à queda.

Avaliação cognitiva e psicossocial

A avaliação cognitiva será realizada com o 10-Point Cognitive Screener (10-CS) (35). Esta ferramenta contempla avaliação de orientação temporal (ano, mês e dias atuais), fluência verbal (nomeação de animais em 1 minuto) e evocação de três palavras (como óculos, caneta e martelo) aprendidas antes da manobra distrativa (35). Os resultados são interpretados da seguinte forma: ≥ 8 pontos compatível com normalidade; 6 a 7 pontos compatível com comprometimento cognitivo possível (geralmente comprometimento cognitivo leve); e 0 a 5 pontos compatível com comprometimento cognitivo provável (geralmente demência) (35). A avaliação de sintomas relacionados a depressão será avaliada pela Escala de Depressão Geriátrica de 15 pontos (GDS-15) (36). Consiste em 15 itens, com possibilidade de resposta de SIM ou NÃO, e pontuação de 0 ou 1 (36). A resposta

será autorrelatada pontuação total superior ou igual a cinco indica a presença de sintomas clinicamente significativos para depressão (36).

- Avaliação de gravidade clínica

Gravidade clínica também será avaliado no projeto através do *National Early Warning Score* (NEWS) 2. Trata-se de escore de alerta precoce com objetivo de reconhecer pacientes com alto risco de deterioração do estado de saúde (37). A pontuação varia entre 0 a 20, sendo o risco clínico classificado como baixo de 0 a 4, baixo-médio de 0 a 4 com presença de escore 3 em um dos parâmetros, médio de 5 a 6 e alto de 7 a 20 (37). Apresenta relação direta com mortalidade em 30 dias, com risco de 5,5% se 0 a 4 pontos, 11,3% se 5 a 6 pontos, 13,3% se 7 a 8 pontos e 27,6% se 9 ou mais pontos (37).

- Avaliação de vulnerabilidade geriátrica e fragilidade

Para avaliação da vulnerabilidade geriátrica será utilizado o escore PRO-AGE, instrumento de aplicação rápida validado utilizado a partir da admissão do paciente idoso no PA (38). Para o modelo preditivo para admissão hospitalar serão coletadas as variáveis de presença de declínio funcional (4 pontos), hospitalização recente (2 pontos), idade avançada (1 ponto), alteração mental aguda (3 pontos), perda de peso (2 pontos) e paciente do sexo masculino (1 ponto). Para o modelo preditivo para internação hospitalar prolongada e óbito intra-hospitalar serão coletadas as variáveis de declínio funcional (2 pontos), hospitalização recente (1 ponto), idade avançada (1 ponto), perda de peso (1 ponto) e fadiga (1 ponto) (38). A avaliação de fragilidade será realizada com a *Clinical Fraulty Scale* (CFS). Sua classificação é dividida nos seguintes níveis: 1- Muito ativo, 2- Ativo, 3- Regular, 4- Vulnerável, 5- Levemente frágil, 6- Moderadamente frágil, 7- Muito frágil, 8- Severamente frágil e 9- Doente terminal (39).

Figura 2: Fluxograma das ações e intervenções propostas:

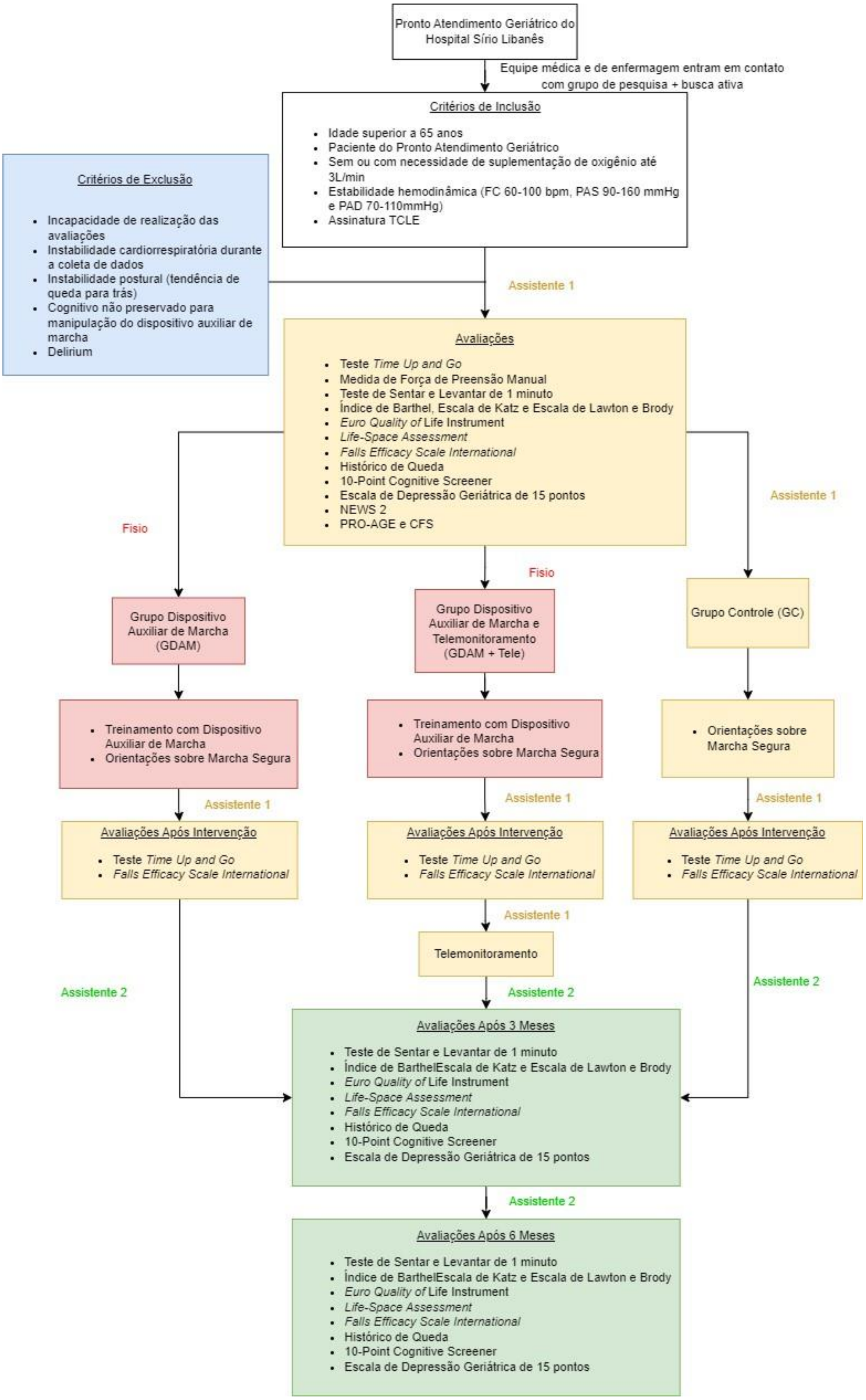
Pronto Atendimento Geriátrico do Hospital Sírio Libanês
Equipe médica e de enfermagem entram em contato com grupo de pesquisa + busca ativa
Critérios de Inclusão
Idade superior a 65 anos
Paciente do Pronto Atendimento Geriátrico
Sem ou com necessidade de suplementação de oxigênio até 3L/min
Estabilidade hemodinâmica (FC 60-100 bpm, PAS 90-160 mmHg e PAD 70-110mmHg)
Assinatura TCLE
Critérios de Exclusão
Incapacidade de realização das avaliações
Instabilidade cardiorrespiratória durante a coleta de dados
Instabilidade postural (tendência de queda para trás)
Cognitivo não preservado para manipulação do dispositivo auxiliar de marcha
Delirium
Assistente 1
Avaliações
Teste Time Up and Go
Medida de Força de Preensão Manual
Teste de Sentar e Levantar de 1 minuto
Índice de Barthel, Escala de Katz e Escala de Lawton e Brody
Euro Quality of Life Instrument
Life-Space Assessment
Falls Efficacy Scale International
Histórico de Queda
10-Point Cognitive Screener
Escala de Depressão Geriátrica de 15 pontos
NEWS 2
PRO-AGE e CFS
Fisio
Fisio
Assistente 1
Grupo Dispositivo Auxiliar de Marcha (GDAM)
Grupo Dispositivo Auxiliar de Marcha e Telemonitoramento (GDAM + Tele)
Grupo Controle (GC)
Treinamento com Dispositivo Auxiliar de Marcha
Orientações sobre Marcha Segura
Treinamento com Dispositivo Auxiliar de Marcha
Orientações sobre Marcha Segura
Orientações sobre Marcha Segura
Assistente 1
Assistente 1
Assistente 1
Avaliações Após Intervenção
Teste Time Up and Go
Falls Efficacy Scale International
Avaliações Após Intervenção
Teste Time Up and Go
Falls Efficacy Scale International
Avaliações Após Intervenção
Teste Time Up and Go
Falls Efficacy Scale International
Assistente 1
Telemonitoramento
Assistente 2
Assistente 2
Assistente 2
Avaliações Após 3 Meses
Teste de Sentar e Levantar de 1 minuto
Índice de BarthelEscala de Katz e Escala de Lawton e Brody
Euro Quality of Life Instrument
Life-Space Assessment
Falls Efficacy Scale International
Histórico de Queda
10-Point Cognitive Screener
Escala de Depressão Geriátrica de 15 pontos
Assistente 2
Avaliações Após 6 Meses
Teste de Sentar e Levantar de 1 minuto
Índice de BarthelEscala de Katz e Escala de Lawton e Brody
Euro Quality of Life Instrument
Life-Space Assessment
Falls Efficacy Scale International
Histórico de Queda
10-Point Cognitive Screener
Escala de Depressão Geriátrica de 15 pontos

Intervenções

As intervenções serão realizadas de acordo com a randomização e após a coleta de dados inicial:

(1) Treinamento com dispositivo auxiliar de marcha: Esse treinamento será realizado nos grupos GADM e GDAM+Tele. Após a coleta de dados inicial, o fisioterapeuta de referência avaliará as necessidades para deslocamento do paciente e fará a indicação do melhor dispositivo. Serão consideradas necessidades relacionadas a cada dispositivo: (A) Bengala: um membro superior para realização da marcha, pequena descarga de peso e necessidade de retorno somatossensorial. A bengala deve ser posicionada entre 15 e 20 cm lateralmente aos pés. A mão do paciente deve ser apoiada na bengala na altura do trocânter maior do fêmur de forma que o cotovelo fique discretamente dobrado, cerca de 30º de flexão. Em geral, utiliza-se a bengala do lado oposto à perna lesionada. (B) Andador: dois membros superiores para realização da marcha, grande descarga de peso e presença de instabilidade postural. Deve-se segurar o equipamento com os braços entre 20 e 25 centímetros à frente do corpo, com os ombros relaxados, tronco ereto, com os cotovelos em flexão de 20° a 30°;

(2)Telemonitoramento: esse acompanhamento será realizado somente no grupo GDAM+Tele. O telemonitoramento ocorrerá a cada duas semanas durante três meses após a alta do PA, será realizado por videochamadas de aproximadamente 15 minutos nas quais serão reforçados a importância do uso do dispositivo auxiliar de marcha e as orientações sobre marcha segura. Na indisponibilidade no primeiro contato, serão realizadas mais duas tentativas nos dias subsequentes.

(3) Orientações sobre marcha segura: as orientações sobre marcha segura serão realizadas para os grupos GDAM, GDAM+Tele e GC. Os pacientes serão orientados com estratégias baseadas em prevenção de quedas (40) e receberão um impresso com as orientações de segurança para as movimentação para se levantar, andar e subir escadas.

Análise estatística

As variáveis contínuas serão expressas em média e desvio padrão (DP) ou mediana e intervalo interquartil (IIQ) 25%-75%. Os dados categóricos serão apresentados em número absoluto e relativo (%). Será aplicado o teste de normalidade Shapiro Wilk. Serão utilizados

os testes t pareado para dados paramétricos ou o teste de Wilcoxon para dados não-paramétricos. Para avaliação entre os três grupos de intervenção serão utilizados o teste One-Way ANOVA para dados paramétricos ou Kruskall-Wallis para dados não-paramétricos. Para avaliar as correlações serão utilizados os testes de correlação de Pearson para dados paramétricos e o teste de Spearman para dados não-paramétricos. Todas as análises serão realizadas utilizando o pacote de estatístico software Statistical Package for Social Sciences (SPSS) versão 28.0.1 (SPSS Inc.®; Chicago, IL, USA), considerando nível de significância de 5%.

Aspectos éticos e aprovação do estudo

Os aspectos éticos deste estudo serão submetidos para avaliação e aprovação do Comitê do Hospital Sírio-Libanês. O estudo será conduzido em conformidade com as resoluções nacionais e internacionais como descritas na Resolução nº 466, de 12 de dezembro de 2012 e na Declaração de Helsinque e todas as suas revisões e alterações. O Termo de Consentimento Livre e Esclarecido (TCLE) será aplicado diretamente ao participante voluntário, atestando a concordância de participação e inclusão no estudo.

Potenciais benefícios e riscos com a realização do estudo

Os potenciais benefícios deste estudo serão a identificação de idosos com alterações de marcha e a realização de uma intervenção com o intuito de aumentar a mobilidade dos idosos e minimizar os riscos de queda. Quanto aos riscos, os participantes voluntários deste estudo poderão relatar diferentes graus de desconforto, cansaço ou fadiga, principalmente durante a execução dos testes funcionais e de força e da aplicação da entrevista. No entanto, estes sintomas deverão cessar após 5 minutos de repouso. Todas as informações coletadas serão compiladas e gerenciadas por meio do sistema de captura de dados eletrônicos (REDCap) hospedada nos servidores do Hospital Sírio-Libanês.

RESULTADOS ESPERADOS

O medo de quedas entre os idosos pode resultar em uma redução significativa de sua mobilidade. Essa apreensão pode levar os idosos a evitar atividades e espaços que considerem arriscados, limitando assim sua participação social e sua capacidade de se

deslocar com autonomia. No entanto, uma intervenção eficaz no pronto atendimento desses idosos envolvendo a disponibilização e o treinamento de dispositivos auxiliares de marcha pode ajudar a reduzir o medo de queda e melhorar sua mobilidade nos ambientes em que vivem. Além disso, o uso do telemonitoramento pode potencializar esses resultados, permitindo um acompanhamento remoto e contínuo da saúde e bem-estar dos idosos, oferecendo suporte adicional e contribuindo para uma maior segurança e confiança no desempenho de suas atividades diárias.

Fonte de fomento

Proposta de Investimento em Desenvolvimento Acadêmico e Científico – Núcleo Avançado de Geriatria (NAGe).

REFERÊNCIAS

1. American College of Emergency Physicians; American Geriatrics Society; Emergency Nurses Association; Society for Academic Emergency Medicine; Geriatric Emergency Department Guidelines Task Force. Geriatric emergency department guidelines. Ann Emerg Med. 2014;63(5):e7-25.

2. Lucke JA, Mooijaart SP, Heeren P, Singler K, McNamara R, Gilbert T, et al. Providing care for older adults in the Emergency Department: expert clinical recommendations from the European Task Force on Geriatric Emergency Medicine. Eur Geriatr Med. 2022;13(2):309-317.

3. James BD, Boyle PA, Buchman AS, Bennett DA. Relation of late-life social activity with incident disability among community-dwelling older adults. J Gerontol A Biol Sci Med Sci. 2011 Apr;66(4):467-73.

4. Brown CJ, Flood KL. Mobility limitation in the older patient: a clinical review. JAMA. 2013;310(11):1168-77.

5. Bateni H, Maki BE. Assistive devices for balance and mobility: benefits, demands, and adverse consequences. Arch Phys Med Rehabil. 2005 Jan;86(1):134-45.

6. Omana H, Madou E, Divine A, Wittich W, Hill KD, Johnson AM, Holmes JD, Hunter SW. The Differential Effect of First-Time Single-Point Cane Use between Healthy Young and Older Adults. PM R. 2021 Dec;13(12):1399-1409.

7. van den Berg N, Schumann M, Kraft K, Hoffmann W. Telemedicine and telecare for older patients--a systematic review. Maturitas. 2012 Oct;73(2):94-114.

8. Merrell RC. Geriatric Telemedicine: Background and Evidence for Telemedicine as a Way to Address the Challenges of Geriatrics. Healthc Inform Res. 2015;21(4):223-9.
9. Batsis JA, DiMilia PR, Seo LM, Fortuna KL, Kennedy MA, Blunt HB, Bagley PJ, Brooks J, Brooks E, Kim SY, Masutani RK, Bruce ML, Bartels SJ. Effectiveness of Ambulatory Telemedicine Care in Older Adults: A Systematic Review. J Am Geriatr Soc. 2019 Aug;67(8):1737-1749.
10. Takahashi PY, Pecina JL, Upatising B, Chaudhry R, Shah ND, Van Houten H, Cha S, Croghan I, Naessens JM, Hanson GJ. A randomized controlled trial of telemonitoring in older adults with multiple health issues to prevent hospitalizations and emergency department visits. Arch Intern Med. 2012 May 28;172(10):773-9.
11. Gellis ZD, Kenaley BL, Ten Have T. Integrated telehealth care for chronic illness and depression in geriatric home care patients: the Integrated Telehealth Education and Activation of Mood (I-TEAM) study. J Am Geriatr Soc. 2014 May;62(5):889-95.
12. Morgan MT, Friscia LA, Whitney SL, Furman JM, Sparto PJ. Reliability and validity of the Falls Efficacy Scale-International (FES-I) in individuals with dizziness and imbalance. Otol Neurotol. 2013 Aug;34(6):1104-8.
13. Kahn JH, Magauran BG Jr, Olshaker JS, Shankar KN. Current Trends in Geriatric Emergency Medicine. Emerg Med Clin North Am. 2016 Aug;34(3):435-52.
14. Organization WH. International Statistical Classification of Diseases and Related Health Problems 10th Revision (ICD-10). F05 Delirium, not induced by alcohol and other psychoactive substances. http://apps.who.int/classifications/icd10/browse/2016/en2016.
15. 13. Inouye SK. Delirium-A Framework to Improve Acute Care for Older Persons. J Am Geriatr Soc. 2018;66(3):446-51.
16. Tinetti ME, Richman D, Powell L. Falls efficacy as a measure of fear of falling. J Gerontol. 1990;45(6):P239-43.
17. França AB, Low G, de Souza Santos G, da Costa Serafim R, Vitorino LM. Psychometric properties of the falls efficacy scale-international and validating the short version among older Brazilians. Geriatr Nurs. 2021;42(2):344-50.
18. Baker PS, Bodner EV, Brown CJ, Kennedy RE, Allman RM. Life-Space Assessment composite score rationale. Clin Rehabil. 2016;30(1):95-7.
19. Simões MDSM, Garcia IF, Costa LDC, Lunardi AC. Life-Space Assessment questionnaire: Novel measurement properties for Brazilian community-dwelling older adults. Geriatr Gerontol Int. 2018 May;18(5):783-789.
20. Barry E, Galvin R, Keogh C, Horgan F, Fahey T. Is the Timed Up and Go test a useful predictor of risk of falls in community dwelling older adults: a systematic review and meta-analysis. BMC Geriatr. 2014;14:14.

21. Bennell K, Dobson F, Hinman R. Measures of physical performance assessments: Self-Paced Walk Test (SPWT), Stair Climb Test (SCT), Six-Minute Walk Test (6MWT), Chair Stand Test (CST), Timed Up & Go (TUG), Sock Test, Lift and Carry Test (LCT), and Car Task. Arthritis Care Res (Hoboken). 2011;63 Suppl 11:S350-70.
22. Rijk JM, Roos PR, Deckx L, van den Akker M, Buntinx F. Prognostic value of handgrip strength in people aged 60 years and older: A systematic review and meta-analysis. Geriatr Gerontol Int. 2016 Jan;16(1):5-20.
23. Desrosiers J, Bravo G, Hébert R, Dutil E. Normative data for grip strength of elderly men and women. Am J Occup Ther. 1995;49(7):637-44.
24. 20. Ozalevli S, Ozden A, Itil O, Akkoclu A. Comparison of the Sit-to-Stand Test with 6 min walk test in patients with chronic obstructive pulmonary disease. Respir Med. 2007;101(2):286-93.
25. Yurkovich M, Avina-Zubieta JA, Thomas J, Gorenchtein M, Lacaille D. A systematic review identifies valid comorbidity indices derived from administrative health data. J Clin Epidemiol. 2015;68(1):3-14.
26. KATZ S, FORD AB, MOSKOWITZ RW, JACKSON BA, JAFFE MW. STUDIES OF ILLNESS IN THE AGED. THE INDEX OF ADL: A STANDARDIZED MEASURE OF BIOLOGICAL AND PSYCHOSOCIAL FUNCTION. JAMA. 1963;185:914-9.
27. Lino VTS, Pereira SRM, Camacho LAB, Ribeiro Filho ST, Buksman S. Adaptação transcultural da Escala de Independência em Atividades da Vida Diária (Escala de Katz). Cad Saúde Pública. 2008;24(1):103–12.
28. Mahoney FI, Barthel DW. Functional evaluation: the Barthel index. Md State Med J. 1965;14: 61-5.
29. Minosso JSM, Amendola F, Alvarenga MRM, Oliveira MA de C. Validação, no Brasil, do Índice de Barthel em idosos atendidos em ambulatórios. Acta paul enferm [Internet]. 2010Mar;23(2):218–23.
30. Loyd C, Markland AD, Zhang Y, Fowler M, Harper S, Wright NC, et al. Prevalence of Hospital-Associated Disability in Older Adults: A Meta-analysis. J Am Med Dir Assoc. 2020;21(4):455-61.e5.
31. Lawton MP, Brody EM. Assessment of older people: self-maintaining and instrumental activities of daily living. Gerontologist. 1969;9(3):179-86.
32. EuroQol Group. EuroQol--a new facility for the measurement of health-related quality of life. Health Policy. 199;16(3):199-208.
33. EQ-5D (Internet). [Accessed: 12 June 2023] Available at: https://euroqol.org/
34. Pasquetti P, Apicella L, Mangone G. Pathogenesis and treatment of falls in elderly. Clin Cases Miner Bone Metab. 2014;11(3):222-5.

35. Apolinario D, Lichtenthaler DG, Magaldi RM, Soares AT, Busse AL, Amaral JR, et al. Using temporal orientation, category fluency, and word recall for detecting cognitive impairment: the 10-point cognitive screener (10-CS). Int J Geriatr Psychiatry. 2016;31(1):4-12.
36. Shin C, Park MH, Lee SH, Ko YH, Kim YK, Han KM, et al. Usefulness of the 15-item geriatric depression scale (GDS-15) for classifying minor and major depressive disorders among community-dwelling elders. J Affect Disord. 2019;259:370-5.
37. Royal College of Physicians. National Early Warning Score (NEWS) 2 [Internet]. Royal College of Physicians. Royal College of Physicians; 2017. Available from: https://www.rcplondon.ac.uk/projects/outputs/national-early-warning-score-news-2.
38. Curiati PK, Gil-Junior LA, Morinaga CV, Ganem F, Curiati JAE, Avelino-Silva TJ. Predicting Hospital Admission and Prolonged Length of Stay in Older Adults in the Emergency Department: The PRO-AGE Scoring System. Ann Emerg Med. 2020;76(3):255-265.
39. Aprahamian I, Cezar NOC, Izbicki R, Lin SM, Paulo DLV, Fattori A, Biella MM, Jacob Filho W, Yassuda MS. Screening for Frailty With the FRAIL Scale: A Comparison With the Phenotype Criteria. J Am Med Dir Assoc. 2017 Jul 1;18(7):592-596.
40. Gillespie LD, Robertson MC, Gillespie WJ, Sherrington C, Gates S, Clemson LM, Lamb SE. Interventions for preventing falls in older people living in the community. Cochrane Database Syst Rev. 2012 Sep 12;2012(9):CD007146.

ENGLISH VERSION

TRAINING AND PROVISION OF MOBILITY AIDS TO PROMOTE AUTONOMY AND MOBILITY OF OLDER PATIENTS IN A GERIATRIC EMERGENCY DEPARTMENT: A PROTOCOL FOR A RANDOMIZED CONTROLLED TRIAL

Research Team:

Pesquisadores responsáveis:

Fernanda Sato Polesel

Sâmia Denadai

Marlon Juliano Romero Aliberti

Christian Valle Morinaga

Mario Chueire de Andrade-Junior

Itiana Cardoso Madalena

Wellington Pereira Yamaguti

Pedro Kallas Curiati

Renato Fraga Righetti

Hospital Sírio-Libanês, São Paulo – SP, Brazil.

ABSTRACT

INTRODUCTION: Older adults have higher rates of emergency department (ED) admissions when compared to their younger counterparts. Mobility is the ability to move around, but also encompasses the environment and the ability to adapt to it. Walking aids can be used to improve mobility and prevent falls. According to international guidelines, they must be available in Geriatric EDs. This study aims to evaluate the efficacy of a program of training and provision of walking aids (WA), associated or not with telemonitoring, on fear of falling, mobility, quality of life and risk of falls up to 3 and 6 months in older adults cared for in an ED. **METHODS:** A randomized clinical trial will be carried out in the ED. Participants will be randomized and allocated into three groups, as follows: A) walking aid group; B) walking aid and telemonitoring group; C) Control group. Patients will undergo a baseline evaluation encompassing sociodemographic and clinical data, mobility in life spaces (Life Space Assessment), gait speed, muscle strength, functionality (Barthel Index, Katz index, and Lawton Scale), quality of life (Euro Quality of Life Instrument-5D), fear of falling (Falls Efficacy Scale International), history of falls, cognition (10-Point Cognitive Screener) and mood (15-point Geriatric Depression Scale) before the intervention. Gait time and fear of falling will be assessed again after the intervention. Finally, mobility in life spaces, functionality, quality of life, fear of falling, history of falls, cognition, and mood will be assessed 3 and 6 months after discharge from the geriatric ED through a telephone interview. For statistical analysis, paired T-test, Wilcoxon test, One-Way ANOVA, Kruskall-Wallis, Pearson correlation, and Spearman test will be used according to the normality established by the Shapiro-Wilk normality test. Results will be considered significant when $P<0.05$.

Keywords: clinical trial, geriatric, mobility, life space, walking aids, emergency room

INTRODUCTION

According to American Geriatric emergency department (ED) Guidelines, proper care for older patients requires policies, protocols, and flows designed for the specific needs of this population [1]. The European geriatric ED guidelines quote as main recommendations for this population the “5 Ms of Geriatrics”: mind, medication, multi-complexity, “most important” and mobility. In other words, during care for older adults in ED, it is recommended: approach to dementia, delirium, depression and cognitive impairment; structured assessment and review of medications used by older adults, considering possible drug interactions and inappropriate use of medication; consideration of general needs of the older adults, as medical, psychological, social, functional or environmental; regarding item “most important” it is recommended ensure the heath results individually focusing on objectives that are meaningful to the older adults [2]. It is also recommended that geriatric ED physical space focus on structural modifications aimed at safety, comfort, memory cues and sensorial perception (both vision and hearing), ensuring greater mobility for older adults. Furthermore, guidelines emphasize that mobility and safety improvement, are not only related to furniture, but also reinforce the importance of easy access to walking aids [1].

Mobility limitations are common in older adults and are associated with depressive symptoms and decrease quality of life, culminate in decrease social interaction, isolation and loneliness, affecting physical, psychological and social aspects of older adults [3]. Risk factors that are more associated with mobility limitation are advanced age, low physical activity, obesity, strength and balance impairment, gait alteration and chronic diseases, in addition to other factors less reported such as depressive symptoms and cognitive impairment, using alcohol or tobacco, recent hospitalization [4]. Therefore, mobility assessment should be considered as a component to be included in older adults heath care [4].

Interventions with walking aids promote independency through biomechanical stabilization, balance and control motor improvement, sensorial feedback, decrease load on the lower limbs, decrease fear falling and fall prevention [5, 6]. Furthermore, several studies point telemonitoring as a tool for health care [7]. It is defined as the use of information technology and telecommunications for remote health care [8]. In a systematic review published in 2019, it was considered viable and well received for older adults heath care and recommended for clinical practice because overcome barriers of distance and access to health services [9]. Few studies have explored telemonitoring for population in ED service, despite evidence that this intervention with older adults with multiple diseases reduce the number of hospitalizations and ED visit [10, 11]. In fact, there is no data on the effects of an intervention encompassing gait devices, associated or not with telemonitoring, to improve the mobility of older patients admitted to the ED.

The hypothesis of the study is that the provision of walking aids enhances mobility in living spaces and will have a significant impact on fear of falling, functionality, and quality of life. Therefore, this study aims to evaluate the efficacy of a program of training and provision of walking aids, associated or not with telemonitoring, on fear falling, mobility, quality of life, and risk of falls up to 3 and 6 months in older adults cared for in an ED.

Objective

Primary objective

Evaluate the efficacy of a program of training and provision of walking aids, associated or not with telemonitoring, on fear falling, mobility and balance in older adults cared for in an ED.

Secondary objective

Evaluate the efficacy of a program of training and provision of walking aids, associated or not with telemonitoring, on quality of life and risk of falls up to 3 and 6 months in older adults cared for in an ED.

METHODS

Study Design

Initially, the amendment to the research project will be submitted to the Research Ethics Committee of Hospital Sírio-Libanês. The protocol of this research project will be registered on "ClinicalTrials.gov" platform as a way of providing information regarding this clinical trial, as well as ensuring transparency in the conduct of stages of this study. This will be a randomized and blind clinical trial. It will be conducted on facilities of Hospital Sírio-Libanês (HSL) – Bela Vista Unit, an institution located in the city of São Paulo, Brazil.

Population

Older adults who will be receiving care in ED of HSL will be recruited.

Sample Size Calculation

Sample size calculation was based on Morgan and cols study [12], which described an important minimum difference of 8,2 points on fear of falling (FES-I), with a standard deviation of 12 points. For an α of 0.05 and a power of 0.80, the total sample size was defined as 42 individuals. Due to the estimated loss of 20% during follow-up, the sample necessary for the success of the study was expanded to 51 individuals in each group, totalizing 153 participants.

Inclusion Criteria

For patients who sign Informed Consent Term, following inclusion criteria will be adopted: aged 65 years or older, admitted to the Geriatric ED of HSL, with at least one criteria of institutional protocol for indication and training of mobility aids: reduction of postural instability; improvement of motor control; increase of somatosensory feedback; reduction of biomechanical overload; safe promotion of autonomy; fall history (in the last six months).

Exclusion Criteria

Exclusion criteria will be: altered level of conscience, need for supplemental oxygen (≥3L/min), respiratory distress, hemodynamic instability, postural instability with a tendency to fall backward, cognitive impairment that limits the use of walking aids, hospitalization after ED evaluation, and delirium.

Randomization and allocation

The process of randomization will be carried out by the software *"Research Electronic Data Capture"* (REDCap), ensuring a similar chance to be allocate to any of intervention groups (1:1:1). Randomization will determine allocation in three groups: walking aid (WA) group; walking aid with telemonitoring (WAT) group; control group.

- Groups

- **Walking aid (WA) group:** patients will be trained for the use of a walking aid and receive guidance on safe gait but will not receive telemonitoring.
- **Walking aid with telemonitoring (WAT) group:** patients will receive training for the use of a walking aid, guidance on safe gait, and telemonitoring.
- **Control group:** patients will receive only guidance on safe gait and will not receive training for the use of walking aid or telemonitoring.

- Blinding

This study will be blind for the researchers involved in the assessments at 3 and 6 months and the researcher who will analyze the data.

Procedures

- Recruitment

Participants will be recruited in the Geriatric ED of HSL, Bela Vista Unit, São Paulo, Brazil, by a trained research assistant, who will remain on duty for 25 hours per week in shifts distributed between 7:00 and 18:00 for up to 6 months. This may be triggered by physician or nurse on duty, but researcher will also actively search for potential candidates for the study. Patients eligible for screening for the study will be contacted by researcher and will signed the Informed Consent Term.

- Screening

Initially, all volunteer participants will undergo a screening process to ensure the eligibility criteria for this study. In this step, the participants are going to be interviewed for data collection (social, demographic, clinical, and medication history). Also, delirium will be considered as an exclusion criteria [13, 14]. For this evaluation will be used Confusion Assessment Method (CAM) [15].

- Assessment Moments

Baseline assessment will be carried out before interventions. Assessment of gait speed and fear of falling will be repeated immediately after the intervention. Fear of falling, life-space mobility, functionality, quality of life, fall history and cognition

assessments will be repeated 3 and 6 months after intervention by a telephone interview or video call.

- Fear of Falling

Fear of falling will be evaluated by Falls Efficacy Scale International (FES-I) [16]. This tool has 16 items that evaluate, for example, walking on a slippery surface or an uneven surface, visiting a friend/relative, or attending a social event [17]. The fear of falling in each activity is classified on a 4-point scale (1 - not at all concerned to 4 - very concerned) [17]. The total score ranges from 16 to 64: 16-22 (low concern), 20-27 (moderate concern), and 28-64 (high concern) [17].

- Mobility in life-space

Mobility in life space will be assessed by the Life Space Assessment (LSA), which allows the characterization of mobility in life-spaces (other rooms of your home besides the room where you sleep; to an area outside your home, places outside your neighborhood but within your town; and to places outside your town), specifically frequency, need for mobility aids and the help of third party in the last 4 weeks [18]. The score reflects the distance, frequency and level of independence, with a range from 0 to 120 [19].

- Gait Assessment

Timed Up and Go test (TUG) evaluate mobility, balance, gait, and risk of falling [20]. In TUG, the researcher requests the participant to stand up on the chair (without using arms), walk 3 meters, go around outside a cone, walk back to the chair, and sit down without using arms [21].

- Peripheral Muscle Strength

Peripheral muscle strength will be measured by handgrip strength (HGS) with a hand-held dynamometer (model SH 5001, brand SAEHAN) [22], respecting the protocol recommended by the American Association of Hand Therapists (ASHT) [23]. The best score between the three measures is going to be considered a handgrip strength measure. For this assessment will be used the dynamometer from the HSL physical therapy research team.

- Functional capacity

One-minute sit-to-stand test will be based on a protocol described by Ozalevli and cols [24]. With the hands on the hip, participants will be guided to sit and stand completely in a chair (height 46 centimeters) as often as possible during 1 minute [24]. To basic activities of daily living (ADL) assessment will be used the Katz Index [25]. As a result, participants will be classified from 0 (independency for ADL) to 6 (total dependency for ADL) [26, 27]. The Barthel index evaluates the autonomy for self-care, in addition to mobility [28-30]. The final score ranges from 0 to 100 and each item is scored according to how the individual performs each task (independently, with some help, or dependently) [28-30]. The higher the score, the higher the independency: 80 to 100 indicate independence; 60-79 points for slight dependency; 40-59 points for moderate independence; 20-39 points for severe dependency; 0-20 points for total dependency [28-30]. Instrumental ADL will be evaluated by the Lawton-Brody scale, which includes seven activities: the ability to use the telephone, mode of transportation, shopping, housekeeping, food preparation, responsibility for own medications, and ability to handle finances [31]. The final score classifies the individuals as independent (25 to 27 points), mild dependent (21 to 25 points), moderate dependent (16 to 20 points), severe dependent (10 to 15 points) and totally dependent (9 points) [31].

- Quality of Life

Quality of life will be evaluated by Euro Quality of Life Instrument-5D (EQ-5D) [32]. This instrument covers five health dimensions: mobility, self-care, usual activities, pain/discomfort, and anxiety/depression [33]. Each dimension is classified into 3 levels: no problems (1 point), some problems (2 points), and extreme problems (3 points) [33]. In the end, the instrument has self-rated health on a visual analog scale ranging from 0 (worst imaginable health state) to 100 (best imaginable health state), resulting in 243/3125 health state distinct [32].

- Fall History

Fall history will be evaluated periodically during the study and the patients must fill out a diary to register every moment in which they fall [34]. For each fall, location, associated injuries, need for special care after the fall will be evaluated.

- Cognition and Mood Assessment

Cognitive assessment will be performed using the 10-Point Cognitive Screener (10-CS) [35]. This tool covers an evaluation of temporal orientation (year, month and day), verbal fluency (naming animals in 1 minute), and three-word recall (as glasses, pen and hammer) learned before the distraction maneuver [35]. Results are interpreted as follows: ≥ 8 points – normality, 6 to 7 points - possible cognitive impairment (usually mild cognitive impairment), and 0 to 5 points - probable cognitive impairment (usually dementia) [35]. Assessment of mood disorders will be evaluated by the Geriatric Depression Scale (GDS-15) [36], which consists of 15 items (possible answers Yes or No) and a score of 0 or 1 [36]. The final score greater or equal to 5 points indicates the presence of significant symptoms of depression [36].

- Geriatric Vulnerability and Frailty Assessment

Geriatric vulnerability will be evaluated by the PRO-AGE scoring system, a fast tool developed to assess older patients in the ED [38]. For the predictive model for hospital admission will be collected variables of presence of functional decline (4 points), recent hospitalization (2 points), advanced age (1 point), acute mental change (3 points), weight loss (2 points) and male patient (1 point). For the predictive model for prolonged hospital stay and in-hospital death will be collected variables of functional decline (2 points), recent hospitalization (1 point), advanced age (1 point), weight loss (1 point) and fatigue (1 point) [38]. Frailty will be evaluated using the Clinical Frailty Scale (CFS). Patients will be classified in one of the following categories: 1- very fit; 2- well; 3- managing well; 4- vulnerable; 5- mildly frail; 6- moderately frail; 7- severely frail; 8- very severely frail; 9- terminally ill [39].

Figure 1. Flow diagram of the clinical trial

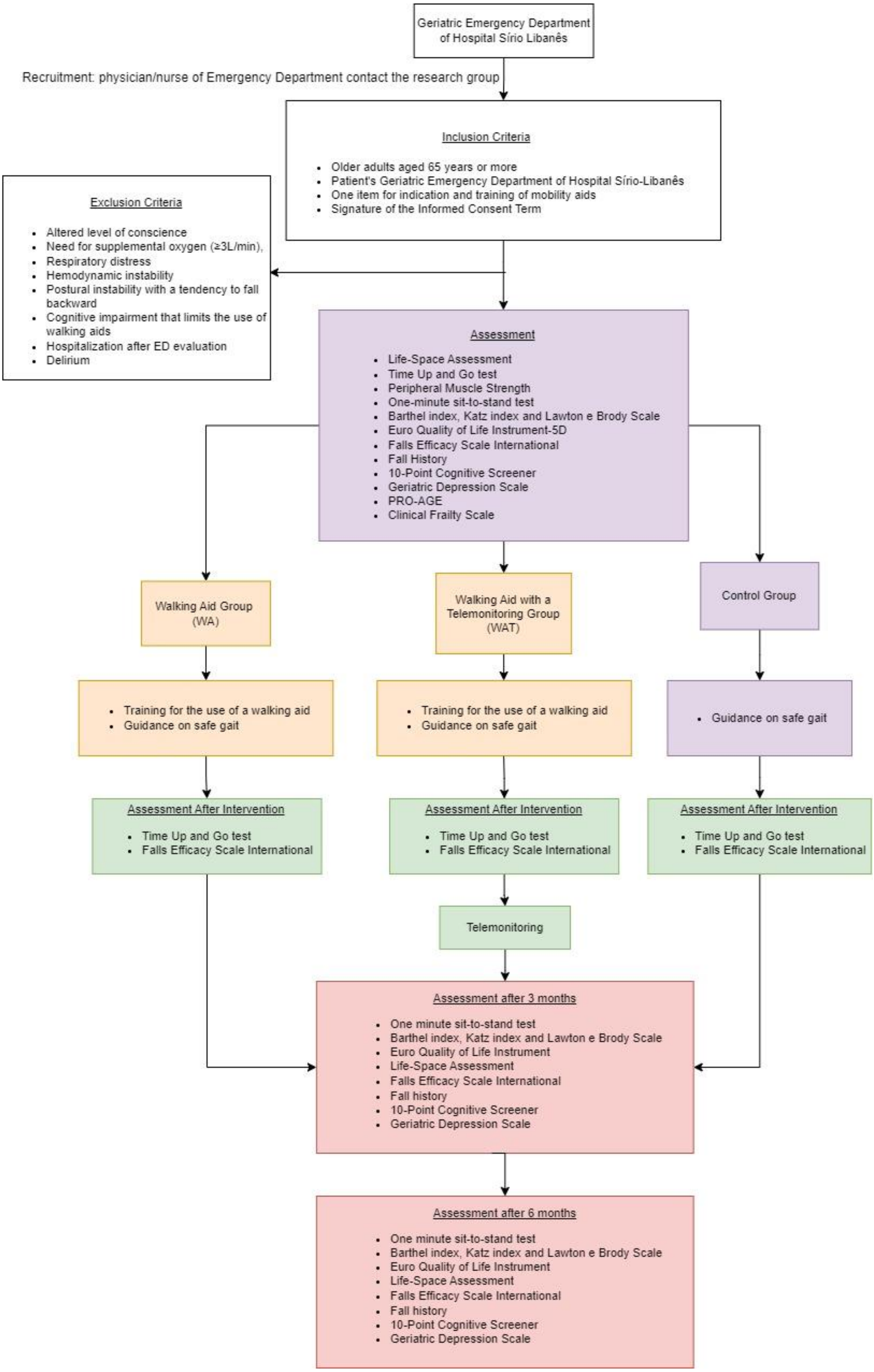

Interventions

The interventions will be performed according to the randomization after the baseline data collection.

1. Training of walking aids

This training will be carried out with patients from groups WA and WAT. After data collection, physical therapist will identify the mobility needs and will indicate the most appropriate walking aid. It will be considered the following needs related for each aid: (A) Canes: one upper limb used for a walk, light weight-bearing and need for somatosensorial feedback; the cane must be positioned between 15 and 20 centimeters laterally to the feet; the patient's hand should be supported on the cane at the height of the greater trochanter of the femur and the elbow should be flexed approximately by 30º; in general, the cane is used on the opposite side of the injured leg. (B) Walkers: both upper limbs used to walk, heavy weight bearing, and presence of postural instability; the equipment must be held between 20 and 25 centimeters in front of the body with relaxed shoulders, erect torso, and elbow flexed at 20º to 30º;

2. *Telemonitoring*

*This f*ollow-up will be carried out only in the WAT group. Telemonitoring will occur every two weeks for three months after the ED discharge, through video call (about 15 minutes), the importance of using mobile devices and the guidance on safe gait will be reinforced. If unavailability on first contact, two more attempts will be made on subsequent days.

3. Guidance on Safe Gait

Guidance on safe gait will be carried out for all the groups in the study. Subjects will be instructed on strategies for fall prevention [40] and will receive a printed leaflet summarizing for getting up, walking and climbing stairs.

Statistical Analysis

Continuous variables will be expressed on average and standard deviation or median and interquartile interval 25%-75%. Categorical data will be presented in absolute and relative numbers. To verify the distribution normality of data, the Shapiro-Wilk normality test will be applied. Paired t-test for parametric data or a Wilcoxon test for non-parametric data will be used. Evaluation between the three intervention groups the One-way ANOVA test for parametric data or Kruskall-Wallis for non-parametric data will be used. To evaluate correlations the Pearson correlation test will be used for parametric data and the Spearman test will be used for non-parametric data. All the analyzes will be carried out using the Statistical Package for Social Sciences (SPSS) version 28.0.1 (SPSS Inc.®; Chicago, IL, USA), considering a significance level of 5%.

Ethics approval and consent to participate

This study received ethical approval from the Research Ethics Committee of the Hospital Sírio-Libanês. The study will be conducted in accordance with national and international resolutions as described in Resolution nº 466 of December 12, 2012 and in the Declaration of Helsinki and all its revisions and amendments. Informed Consent Form will be applied directly to the voluntary participant, certifying agreement to participate and be included in the study.

Potential benefits and risks of carrying out the study

Potential benefits of this study will be identification of older adults with gait changes and implementation of an intervention with the aim of increasing the mobility of older adults and minimizing the risk of falling. Regarding risks, volunteer participants in this study may report different degrees of discomfort, tiredness or fatigue, especially

during performance of functional and strength tests. However, these symptoms should cease after 5 minutes of rest. All information collected will be compiled and managed through the electronic data capture system (REDCap) hosted on the servers of Hospital Sírio-Libanês.

Expected Results

Fear of falls among older adults can result in a significant reduction in their mobility. This apprehension can lead older adults to avoid activities and spaces that they consider risky, thus limiting their social participation and their ability to move independently. However, an effective intervention in the ED of these older adult involving the provision and training of walking aids can help reduce their fear of falling and improve their mobility in the environment. Furthermore, the use of telemonitoring can enhance these results, allowing remote and continuous monitoring of the health and well-being of older adults, offering additional support and contributing to greater security and confidence in carrying out their daily activities.

Funding

The study was funded by the Geriatric Center for Advanced Medicine, Hospital Sírio-Libanês, São Paulo.

References

1. American College of Emergency Physicians; American Geriatrics Society; Emergency Nurses Association; Society for Academic Emergency Medicine; Geriatric Emergency Department Guidelines Task Force. Geriatric emergency department guidelines. Ann Emerg Med. 2014;63(5):e7-25.

2. Lucke JA, Mooijaart SP, Heeren P, Singler K, McNamara R, Gilbert T, et al. Providing care for older adults in the Emergency Department: expert clinical recommendations from the European Task Force on Geriatric Emergency Medicine. Eur Geriatr Med. 2022;13(2):309-317.

3. James BD, Boyle PA, Buchman AS, Bennett DA. Relation of late-life social activity with incident disability among community-dwelling older adults. J Gerontol A Biol Sci Med Sci. 2011 Apr;66(4):467-73.

4. Brown CJ, Flood KL. Mobility limitation in the older patient: a clinical review. JAMA. 2013;310(11):1168-77.

5. Bateni H, Maki BE. Assistive devices for balance and mobility: benefits, demands, and adverse consequences. Arch Phys Med Rehabil. 2005 Jan;86(1):134-45.

6. Omana H, Madou E, Divine A, Wittich W, Hill KD, Johnson AM, Holmes JD, Hunter SW. The Differential Effect of First-Time Single-Point Cane Use between Healthy Young and Older Adults. PM R. 2021 Dec;13(12):1399-1409.

7. van den Berg N, Schumann M, Kraft K, Hoffmann W. Telemedicine and telecare for older patients--a systematic review. Maturitas. 2012 Oct;73(2):94-114.

8. Merrell RC. Geriatric Telemedicine: Background and Evidence for Telemedicine as a Way to Address the Challenges of Geriatrics. Healthc Inform Res. 2015;21(4):223-9.

9. Batsis JA, DiMilia PR, Seo LM, Fortuna KL, Kennedy MA, Blunt HB, Bagley PJ, Brooks J, Brooks E, Kim SY, Masutani RK, Bruce ML, Bartels SJ. Effectiveness of Ambulatory Telemedicine Care in Older Adults: A Systematic Review. J Am Geriatr Soc. 2019 Aug;67(8):1737-1749.

10. Takahashi PY, Pecina JL, Upatising B, Chaudhry R, Shah ND, Van Houten H, Cha S, Croghan I, Naessens JM, Hanson GJ. A randomized controlled trial of telemonitoring in older adults with multiple health issues to prevent hospitalizations and emergency department visits. Arch Intern Med. 2012 May 28;172(10):773-9.

11. Gellis ZD, Kenaley BL, Ten Have T. Integrated telehealth care for chronic illness and depression in geriatric home care patients: the Integrated Telehealth Education and Activation of Mood (I-TEAM) study. J Am Geriatr Soc. 2014 May;62(5):889-95.

12. Morgan MT, Friscia LA, Whitney SL, Furman JM, Sparto PJ. Reliability and validity of the Falls Efficacy Scale-International (FES-I) in individuals with dizziness and imbalance. Otol Neurotol. 2013 Aug;34(6):1104-8.

13. Kahn JH, Magauran BG Jr, Olshaker JS, Shankar KN. Current Trends in Geriatric Emergency Medicine. Emerg Med Clin North Am. 2016 Aug;34(3):435-52.

14. Organization WH. International Statistical Classification of Diseases and Related Health Problems 10th Revision (ICD-10). F05 Delirium, not induced by alcohol and other

psychoactive substances. http://apps.who.int/classifications/icd10/browse/2016/en2016.

15. 13. Inouye SK. Delirium-A Framework to Improve Acute Care for Older Persons. J Am Geriatr Soc. 2018;66(3):446-51.

16. Tinetti ME, Richman D, Powell L. Falls efficacy as a measure of fear of falling. J Gerontol. 1990;45(6):P239-43.

17. França AB, Low G, de Souza Santos G, da Costa Serafim R, Vitorino LM. Psychometric properties of the falls efficacy scale-international and validating the short version among older Brazilians. Geriatr Nurs. 2021;42(2):344-50.

18. Baker PS, Bodner EV, Brown CJ, Kennedy RE, Allman RM. Life-Space Assessment composite score rationale. Clin Rehabil. 2016;30(1):95-7.

19. Simões MDSM, Garcia IF, Costa LDC, Lunardi AC. Life-Space Assessment questionnaire: Novel measurement properties for Brazilian community-dwelling older adults. Geriatr Gerontol Int. 2018 May;18(5):783-789.

20. Barry E, Galvin R, Keogh C, Horgan F, Fahey T. Is the Timed Up and Go test a useful predictor of risk of falls in community dwelling older adults: a systematic review and meta-analysis. BMC Geriatr. 2014;14:14.

21. Bennell K, Dobson F, Hinman R. Measures of physical performance assessments: Self-Paced Walk Test (SPWT), Stair Climb Test (SCT), Six-Minute Walk Test (6MWT), Chair Stand Test (CST), Timed Up & Go (TUG), Sock Test, Lift and Carry Test (LCT), and Car Task. Arthritis Care Res (Hoboken). 2011;63 Suppl 11:S350-70.

22. Rijk JM, Roos PR, Deckx L, van den Akker M, Buntinx F. Prognostic value of handgrip strength in people aged 60 years and older: A systematic review and meta-analysis. Geriatr Gerontol Int. 2016 Jan;16(1):5-20.

23. Desrosiers J, Bravo G, Hébert R, Dutil E. Normative data for grip strength of elderly men and women. Am J Occup Ther. 1995;49(7):637-44.

24. 20. Ozalevli S, Ozden A, Itil O, Akkoclu A. Comparison of the Sit-to-Stand Test with 6 min walk test in patients with chronic obstructive pulmonary disease. Respir Med. 2007;101(2):286-93.

25. Yurkovich M, Avina-Zubieta JA, Thomas J, Gorenchtein M, Lacaille D. A systematic review identifies valid comorbidity indices derived from administrative health data. J Clin Epidemiol. 2015;68(1):3-14.

26. KATZ S, FORD AB, MOSKOWITZ RW, JACKSON BA, JAFFE MW. STUDIES OF ILLNESS IN THE AGED. THE INDEX OF ADL: A STANDARDIZED MEASURE OF BIOLOGICAL AND PSYCHOSOCIAL FUNCTION. JAMA. 1963;185:914-9.

27. Lino VTS, Pereira SRM, Camacho LAB, Ribeiro Filho ST, Buksman S. Adaptação transcultural da Escala de Independência em Atividades da Vida Diária (Escala de Katz). Cad Saúde Pública. 2008;24(1):103–12.

28. Mahoney FI, Barthel DW. Functional evaluation: the Barthel index. Md State Med J. 1965;14: 61-5.

29. Minosso JSM, Amendola F, Alvarenga MRM, Oliveira MA de C. Validação, no Brasil, do Índice de Barthel em idosos atendidos em ambulatórios. Acta paul enferm [Internet]. 2010Mar;23(2):218–23.

30. Loyd C, Markland AD, Zhang Y, Fowler M, Harper S, Wright NC, et al. Prevalence of Hospital-Associated Disability in Older Adults: A Meta-analysis. J Am Med Dir Assoc. 2020;21(4):455-61.e5.

31. Lawton MP, Brody EM. Assessment of older people: self-maintaining and instrumental activities of daily living. Gerontologist. 1969;9(3):179-86.

32. EuroQol Group. EuroQol--a new facility for the measurement of health-related quality of life. Health Policy. 199;16(3):199-208.

33. EQ-5D (Internet). [Accessed: 12 June 2023] Available at: https://euroqol.org/

34. Pasquetti P, Apicella L, Mangone G. Pathogenesis and treatment of falls in elderly. Clin Cases Miner Bone Metab. 2014;11(3):222-5.

35. Apolinario D, Lichtenthaler DG, Magaldi RM, Soares AT, Busse AL, Amaral JR, et al. Using temporal orientation, category fluency, and word recall for detecting cognitive impairment: the 10-point cognitive screener (10-CS). Int J Geriatr Psychiatry. 2016;31(1):4-12.

36. Shin C, Park MH, Lee SH, Ko YH, Kim YK, Han KM, et al. Usefulness of the 15-item geriatric depression scale (GDS-15) for classifying minor and major depressive disorders among community-dwelling elders. J Affect Disord. 2019;259:370-5.

37. Royal College of Physicians. National Early Warning Score (NEWS) 2 [Internet]. Royal College of Physicians. Royal College of Physicians; 2017. Available from: https://www.rcplondon.ac.uk/projects/outputs/national-early-warning-score-news-2.

38. Curiati PK, Gil-Junior LA, Morinaga CV, Ganem F, Curiati JAE, Avelino-Silva TJ. Predicting Hospital Admission and Prolonged Length of Stay in Older Adults in the Emergency Department: The PRO-AGE Scoring System. Ann Emerg Med. 2020;76(3):255-265.

39. Aprahamian I, Cezar NOC, Izbicki R, Lin SM, Paulo DLV, Fattori A, Biella MM, Jacob Filho W, Yassuda MS. Screening for Frailty With the FRAIL Scale: A Comparison With the Phenotype Criteria. J Am Med Dir Assoc. 2017 Jul 1;18(7):592-596.

40. Gillespie LD, Robertson MC, Gillespie WJ, Sherrington C, Gates S, Clemson LM, Lamb SE. Interventions for preventing falls in older people living in the community. Cochrane Database Syst Rev. 2012 Sep 12;2012(9):CD007146.
